# Search for direct top squark pair production in final states with two leptons in $\sqrt{s} = 13$ TeV *pp* collisions with the ATLAS detector

**DOI:** 10.1140/epjc/s10052-017-5445-x

**Published:** 2017-12-22

**Authors:** M. Aaboud, G. Aad, B. Abbott, O. Abdinov, B. Abeloos, S. H. Abidi, O. S. AbouZeid, N. L. Abraham, H. Abramowicz, H. Abreu, R. Abreu, Y. Abulaiti, B. S. Acharya, S. Adachi, L. Adamczyk, J. Adelman, M. Adersberger, T. Adye, A. A. Affolder, Y. Afik, T. Agatonovic-Jovin, C. Agheorghiesei, J. A. Aguilar-Saavedra, S. P. Ahlen, F. Ahmadov, G. Aielli, S. Akatsuka, H. Akerstedt, T. P. A. Åkesson, E. Akilli, A. V. Akimov, G. L. Alberghi, J. Albert, P. Albicocco, M. J. AlconadaVerzini, S. C. Alderweireldt, M. Aleksa, I. N. Aleksandrov, C. Alexa, G. Alexander, T. Alexopoulos, M. Alhroob, B. Ali, M. Aliev, G. Alimonti, J. Alison, S. P. Alkire, B. M. M. Allbrooke, B. W. Allen, P. P. Allport, A. Aloisio, A. Alonso, F. Alonso, C. Alpigiani, A. A. Alshehri, M. I. Alstaty, B. AlvarezGonzalez, D. ÁlvarezPiqueras, M. G. Alviggi, B. T. Amadio, Y. AmaralCoutinho, C. Amelung, D. Amidei, S. P. AmorDosSantos, S. Amoroso, G. Amundsen, C. Anastopoulos, L. S. Ancu, N. Andari, T. Andeen, C. F. Anders, J. K. Anders, K. J. Anderson, A. Andreazza, V. Andrei, S. Angelidakis, I. Angelozzi, A. Angerami, A. V. Anisenkov, N. Anjos, A. Annovi, C. Antel, M. Antonelli, A. Antonov, D. J. Antrim, F. Anulli, M. Aoki, L. AperioBella, G. Arabidze, Y. Arai, J. P. Araque, V. AraujoFerraz, A. T. H. Arce, R. E. Ardell, F. A. Arduh, J.-F. Arguin, S. Argyropoulos, M. Arik, A. J. Armbruster, L. J. Armitage, O. Arnaez, H. Arnold, M. Arratia, O. Arslan, A. Artamonov, G. Artoni, S. Artz, S. Asai, N. Asbah, A. Ashkenazi, L. Asquith, K. Assamagan, R. Astalos, M. Atkinson, N. B. Atlay, K. Augsten, G. Avolio, B. Axen, M. K. Ayoub, G. Azuelos, A. E. Baas, M. J. Baca, H. Bachacou, K. Bachas, M. Backes, P. Bagnaia, M. Bahmani, H. Bahrasemani, J. T. Baines, M. Bajic, O. K. Baker, E. M. Baldin, P. Balek, F. Balli, W. K. Balunas, E. Banas, A. Bandyopadhyay, Sw. Banerjee, A. A. E. Bannoura, L. Barak, E. L. Barberio, D. Barberis, M. Barbero, T. Barillari, M-S. Barisits, J. T. Barkeloo, T. Barklow, N. Barlow, S. L. Barnes, B. M. Barnett, R. M. Barnett, Z. Barnovska-Blenessy, A. Baroncelli, G. Barone, A. J. Barr, L. Barranco Navarro, F. Barreiro, J. Barreiro Guimarães da Costa, R. Bartoldus, A. E. Barton, P. Bartos, A. Basalaev, A. Bassalat, R. L. Bates, S. J. Batista, J. R. Batley, M. Battaglia, M. Bauce, F. Bauer, H. S. Bawa, J. B. Beacham, M. D. Beattie, T. Beau, P. H. Beauchemin, P. Bechtle, H. P. Beck, H. C. Beck, K. Becker, M. Becker, C. Becot, A. J. Beddall, A. Beddall, V. A. Bednyakov, M. Bedognetti, C. P. Bee, T. A. Beermann, M. Begalli, M. Begel, J. K. Behr, A. S. Bell, G. Bella, L. Bellagamba, A. Bellerive, M. Bellomo, K. Belotskiy, O. Beltramello, N. L. Belyaev, O. Benary, D. Benchekroun, M. Bender, K. Bendtz, N. Benekos, Y. Benhammou, E. Benhar Noccioli, J. Benitez, D. P. Benjamin, M. Benoit, J. R. Bensinger, S. Bentvelsen, L. Beresford, M. Beretta, D. Berge, E. Bergeaas Kuutmann, N. Berger, J. Beringer, S. Berlendis, N. R. Bernard, G. Bernardi, C. Bernius, F. U. Bernlochner, T. Berry, P. Berta, C. Bertella, G. Bertoli, F. Bertolucci, I. A. Bertram, C. Bertsche, D. Bertsche, G. J. Besjes, O. Bessidskaia Bylund, M. Bessner, N. Besson, A. Bethani, S. Bethke, A. J. Bevan, J. Beyer, R. M. Bianchi, O. Biebel, D. Biedermann, R. Bielski, K. Bierwagen, N. V. Biesuz, M. Biglietti, T. R. V. Billoud, H. Bilokon, M. Bindi, A. Bingul, C. Bini, S. Biondi, T. Bisanz, C. Bittrich, D. M. Bjergaard, J. E. Black, K. M. Black, R. E. Blair, T. Blazek, I. Bloch, C. Blocker, A. Blue, W. Blum, U. Blumenschein, S. Blunier, G. J. Bobbink, V. S. Bobrovnikov, S. S. Bocchetta, A. Bocci, C. Bock, M. Boehler, D. Boerner, D. Bogavac, A. G. Bogdanchikov, C. Bohm, V. Boisvert, P. Bokan, T. Bold, A. S. Boldyrev, A. E. Bolz, M. Bomben, M. Bona, M. Boonekamp, A. Borisov, G. Borissov, J. Bortfeldt, D. Bortoletto, V. Bortolotto, D. Boscherini, M. Bosman, J. D. Bossio Sola, J. Boudreau, J. Bouffard, E. V. Bouhova-Thacker, D. Boumediene, C. Bourdarios, S. K. Boutle, A. Boveia, J. Boyd, I. R. Boyko, J. Bracinik, A. Brandt, G. Brandt, O. Brandt, U. Bratzler, B. Brau, J. E. Brau, W. D. Breaden Madden, K. Brendlinger, A. J. Brennan, L. Brenner, R. Brenner, S. Bressler, D. L. Briglin, T. M. Bristow, D. Britton, D. Britzger, F. M. Brochu, I. Brock, R. Brock, G. Brooijmans, T. Brooks, W. K. Brooks, J. Brosamer, E. Brost, J. H Broughton, P. A. Bruckman de Renstrom, D. Bruncko, A. Bruni, G. Bruni, L. S. Bruni, S. Bruno, BH Brunt, M. Bruschi, N. Bruscino, P. Bryant, L. Bryngemark, T. Buanes, Q. Buat, P. Buchholz, A. G. Buckley, I. A. Budagov, F. Buehrer, M. K. Bugge, O. Bulekov, D. Bullock, T. J. Burch, S. Burdin, C. D. Burgard, A. M. Burger, B. Burghgrave, K. Burka, S. Burke, I. Burmeister, J. T. P. Burr, E. Busato, D. Büscher, V. Büscher, P. Bussey, J. M. Butler, C. M. Buttar, J. M. Butterworth, P. Butti, W. Buttinger, A. Buzatu, A. R. Buzykaev, S. Cabrera Urbán, D. Caforio, V. M. Cairo, O. Cakir, N. Calace, P. Calafiura, A. Calandri, G. Calderini, P. Calfayan, G. Callea, L. P. Caloba, S. Calvente Lopez, D. Calvet, S. Calvet, T. P. Calvet, R. Camacho Toro, S. Camarda, P. Camarri, D. Cameron, R. Caminal Armadans, C. Camincher, S. Campana, M. Campanelli, A. Camplani, A. Campoverde, V. Canale, M. Cano Bret, J. Cantero, T. Cao, M. D. M. Capeans Garrido, I. Caprini, M. Caprini, M. Capua, R. M. Carbone, R. Cardarelli, F. Cardillo, I. Carli, T. Carli, G. Carlino, B. T. Carlson, L. Carminati, R. M. D. Carney, S. Caron, E. Carquin, S. Carrá, G. D. Carrillo-Montoya, D. Casadei, M. P. Casado, M. Casolino, D. W. Casper, R. Castelijn, V. Castillo Gimenez, N. F. Castro, A. Catinaccio, J. R. Catmore, A. Cattai, J. Caudron, V. Cavaliere, E. Cavallaro, D. Cavalli, M. Cavalli-Sforza, V. Cavasinni, E. Celebi, F. Ceradini, L. Cerda Alberich, A. S. Cerqueira, A. Cerri, L. Cerrito, F. Cerutti, A. Cervelli, S. A. Cetin, A. Chafaq, D. Chakraborty, S. K. Chan, W. S. Chan, Y. L. Chan, P. Chang, J. D. Chapman, D. G. Charlton, C. C. Chau, C. A. Chavez Barajas, S. Che, S. Cheatham, A. Chegwidden, S. Chekanov, S. V. Chekulaev, G. A. Chelkov, M. A. Chelstowska, C. Chen, H. Chen, J. Chen, S. Chen, S. Chen, X. Chen, Y. Chen, H. C. Cheng, H. J. Cheng, A. Cheplakov, E. Cheremushkina, R. Cherkaoui El Moursli, E. Cheu, K. Cheung, L. Chevalier, V. Chiarella, G. Chiarelli, G. Chiodini, A. S. Chisholm, A. Chitan, Y. H. Chiu, M. V. Chizhov, K. Choi, A. R. Chomont, S. Chouridou, Y. S. Chow, V. Christodoulou, M. C. Chu, J. Chudoba, A. J. Chuinard, J. J. Chwastowski, L. Chytka, A. K. Ciftci, D. Cinca, V. Cindro, I. A. Cioara, C. Ciocca, A. Ciocio, F. Cirotto, Z. H. Citron, M. Citterio, M. Ciubancan, A. Clark, B. L. Clark, M. R. Clark, P. J. Clark, R. N. Clarke, C. Clement, Y. Coadou, M. Cobal, A. Coccaro, J. Cochran, L. Colasurdo, B. Cole, A. P. Colijn, J. Collot, T. Colombo, P. Conde Muiño, E. Coniavitis, S. H. Connell, I. A. Connelly, S. Constantinescu, G. Conti, F. Conventi, M. Cooke, A. M. Cooper-Sarkar, F. Cormier, K. J. R. Cormier, M. Corradi, F. Corriveau, A. Cortes-Gonzalez, G. Cortiana, G. Costa, M. J. Costa, D. Costanzo, G. Cottin, G. Cowan, B. E. Cox, K. Cranmer, S. J. Crawley, R. A. Creager, G. Cree, S. Crépé-Renaudin, F. Crescioli, W. A. Cribbs, M. Cristinziani, V. Croft, G. Crosetti, A. Cueto, T. Cuhadar Donszelmann, A. R. Cukierman, J. Cummings, M. Curatolo, J. Cúth, S. Czekierda, P. Czodrowski, G. D’amen, S. D’Auria, L. D’eramo, M. D’Onofrio, M. J. Da Cunha Sargedas De Sousa, C. Da Via, W. Dabrowski, T. Dado, T. Dai, O. Dale, F. Dallaire, C. Dallapiccola, M. Dam, J. R. Dandoy, M. F. Daneri, N. P. Dang, A. C. Daniells, N. S. Dann, M. Danninger, M. Dano Hoffmann, V. Dao, G. Darbo, S. Darmora, J. Dassoulas, A. Dattagupta, T. Daubney, W. Davey, C. David, T. Davidek, D. R. Davis, P. Davison, E. Dawe, I. Dawson, K. De, R. de Asmundis, A. De Benedetti, S. De Castro, S. De Cecco, N. De Groot, P. de Jong, H. De la Torre, F. De Lorenzi, A. De Maria, D. De Pedis, A. De Salvo, U. De Sanctis, A. De Santo, K. De Vasconcelos Corga, J. B. De Vivie De Regie, R. Debbe, C. Debenedetti, D. V. Dedovich, N. Dehghanian, I. Deigaard, M. Del Gaudio, J. Del Peso, D. Delgove, F. Deliot, C. M. Delitzsch, A. Dell’Acqua, L. Dell’Asta, M. Dell’Orso, M. Della Pietra, D. della Volpe, M. Delmastro, C. Delporte, P. A. Delsart, D. A. DeMarco, S. Demers, M. Demichev, A. Demilly, S. P. Denisov, D. Denysiuk, D. Derendarz, J. E. Derkaoui, F. Derue, P. Dervan, K. Desch, C. Deterre, K. Dette, M. R. Devesa, P. O. Deviveiros, A. Dewhurst, S. Dhaliwal, F. A. Di Bello, A. Di Ciaccio, L. Di Ciaccio, W. K. Di Clemente, C. Di Donato, A. Di Girolamo, B. Di Girolamo, B. Di Micco, R. Di Nardo, K. F. Di Petrillo, A. Di Simone, R. Di Sipio, D. Di Valentino, C. Diaconu, M. Diamond, F. A. Dias, M. A. Diaz, E. B. Diehl, J. Dietrich, S. Díez Cornell, A. Dimitrievska, J. Dingfelder, P. Dita, S. Dita, F. Dittus, F. Djama, T. Djobava, J. I. Djuvsland, M. A. B. do Vale, D. Dobos, M. Dobre, C. Doglioni, J. Dolejsi, Z. Dolezal, M. Donadelli, S. Donati, P. Dondero, J. Donini, J. Dopke, A. Doria, M. T. Dova, A. T. Doyle, E. Drechsler, M. Dris, Y. Du, J. Duarte-Campderros, A. Dubreuil, E. Duchovni, G. Duckeck, A. Ducourthial, O. A. Ducu, D. Duda, A. Dudarev, A. Chr. Dudder, E. M. Duffield, L. Duflot, M. Dührssen, C. Dulsen, M. Dumancic, A. E. Dumitriu, A. K. Duncan, M. Dunford, H. Duran Yildiz, M. Düren, A. Durglishvili, D. Duschinger, B. Dutta, D. Duvnjak, M. Dyndal, B. S. Dziedzic, C. Eckardt, K. M. Ecker, R. C. Edgar, T. Eifert, G. Eigen, K. Einsweiler, T. Ekelof, M. El Kacimi, R. El Kosseifi, V. Ellajosyula, M. Ellert, S. Elles, F. Ellinghaus, A. A. Elliot, N. Ellis, J. Elmsheuser, M. Elsing, D. Emeliyanov, Y. Enari, O. C. Endner, J. S. Ennis, J. Erdmann, A. Ereditato, M. Ernst, S. Errede, M. Escalier, C. Escobar, B. Esposito, O. Estrada Pastor, A. I. Etienvre, E. Etzion, H. Evans, A. Ezhilov, M. Ezzi, F. Fabbri, L. Fabbri, G. Facini, R. M. Fakhrutdinov, S. Falciano, R. J. Falla, J. Faltova, Y. Fang, M. Fanti, A. Farbin, A. Farilla, C. Farina, E. M. Farina, T. Farooque, S. Farrell, S. M. Farrington, P. Farthouat, F. Fassi, P. Fassnacht, D. Fassouliotis, M. Faucci Giannelli, A. Favareto, W. J. Fawcett, L. Fayard, O. L. Fedin, W. Fedorko, S. Feigl, L. Feligioni, C. Feng, E. J. Feng, H. Feng, M. J. Fenton, A. B. Fenyuk, L. Feremenga, P. Fernandez Martinez, S. Fernandez Perez, J. Ferrando, A. Ferrari, P. Ferrari, R. Ferrari, D. E. Ferreirade Lima, A. Ferrer, D. Ferrere, C. Ferretti, F. Fiedler, A. Filipčič, M. Filipuzzi, F. Filthaut, M. Fincke-Keeler, K. D. Finelli, M. C. N. Fiolhais, L. Fiorini, A. Fischer, C. Fischer, J. Fischer, W. C. Fisher, N. Flaschel, I. Fleck, P. Fleischmann, R. R. M. Fletcher, T. Flick, B. M. Flierl, L. R. Flores Castillo, M. J. Flowerdew, G. T. Forcolin, A. Formica, F. A. Förster, A. Forti, A. G. Foster, D. Fournier, H. Fox, S. Fracchia, P. Francavilla, M. Franchini, S. Franchino, D. Francis, L. Franconi, M. Franklin, M. Frate, M. Fraternali, D. Freeborn, S. M. Fressard-Batraneanu, B. Freund, D. Froidevaux, J. A. Frost, C. Fukunaga, T. Fusayasu, J. Fuster, C. Gabaldon, O. Gabizon, A. Gabrielli, A. Gabrielli, G. P. Gach, S. Gadatsch, S. Gadomski, G. Gagliardi, L. G. Gagnon, C. Galea, B. Galhardo, E. J. Gallas, B. J. Gallop, P. Gallus, G. Galster, K. K. Gan, S. Ganguly, Y. Gao, Y. S. Gao, F. M. Garay Walls, C. García, J. E. García Navarro, J. A. García Pascual, M. Garcia-Sciveres, R. W. Gardner, N. Garelli, V. Garonne, A. Gascon Bravo, K. Gasnikova, C. Gatti, A. Gaudiello, G. Gaudio, I. L. Gavrilenko, C. Gay, G. Gaycken, E. N. Gazis, C. N. P. Gee, J. Geisen, M. Geisen, M. P. Geisler, K. Gellerstedt, C. Gemme, M. H. Genest, C. Geng, S. Gentile, C. Gentsos, S. George, D. Gerbaudo, A. Gershon, G. Geßner, S. Ghasemi, M. Ghneimat, B. Giacobbe, S. Giagu, N. Giangiacomi, P. Giannetti, S. M. Gibson, M. Gignac, M. Gilchriese, D. Gillberg, G. Gilles, D. M. Gingrich, M. P. Giordani, F. M. Giorgi, P. F. Giraud, P. Giromini, G. Giugliarelli, D. Giugni, F. Giuli, C. Giuliani, M. Giulini, B. K. Gjelsten, S. Gkaitatzis, I. Gkialas, E. L. Gkougkousis, P. Gkountoumis, L. K. Gladilin, C. Glasman, J. Glatzer, P. C. F. Glaysher, A. Glazov, M. Goblirsch-Kolb, J. Godlewski, S. Goldfarb, T. Golling, D. Golubkov, A. Gomes, R. Gonçalo, R. GoncalvesGama, J. Goncalves Pinto Firmino Da Costa, G. Gonella, L. Gonella, A. Gongadze, S. González de la Hoz, S. Gonzalez-Sevilla, L. Goossens, P. A. Gorbounov, H. A. Gordon, I. Gorelov, B. Gorini, E. Gorini, A. Gorišek, A. T. Goshaw, C. Gössling, M. I. Gostkin, C. A. Gottardo, C. R. Goudet, D. Goujdami, A. G. Goussiou, N. Govender, E. Gozani, L. Graber, I. Grabowska-Bold, P. O. J. Gradin, J. Gramling, E. Gramstad, S. Grancagnolo, V. Gratchev, P. M. Gravila, C. Gray, H. M. Gray, Z. D. Greenwood, C. Grefe, K. Gregersen, I. M. Gregor, P. Grenier, K. Grevtsov, J. Griffiths, A. A. Grillo, K. Grimm, S. Grinstein, Ph. Gris, J.-F. Grivaz, S. Groh, E. Gross, J. Grosse-Knetter, G. C. Grossi, Z. J. Grout, A. Grummer, L. Guan, W. Guan, J. Guenther, F. Guescini, D. Guest, O. Gueta, B. Gui, E. Guido, T. Guillemin, S. Guindon, U. Gul, C. Gumpert, J. Guo, W. Guo, Y. Guo, R. Gupta, S. Gupta, G. Gustavino, B. J. Gutelman, P. Gutierrez, N. G. Gutierrez Ortiz, C. Gutschow, C. Guyot, M. P. Guzik, C. Gwenlan, C. B. Gwilliam, A. Haas, C. Haber, H. K. Hadavand, N. Haddad, A. Hadef, S. Hageböck, M. Hagihara, H. Hakobyan, M. Haleem, J. Haley, G. Halladjian, G. D. Hallewell, K. Hamacher, P. Hamal, K. Hamano, A. Hamilton, G. N. Hamity, P. G. Hamnett, L. Han, S. Han, K. Hanagaki, K. Hanawa, M. Hance, B. Haney, P. Hanke, J. B. Hansen, J. D. Hansen, M. C. Hansen, P. H. Hansen, K. Hara, A. S. Hard, T. Harenberg, F. Hariri, S. Harkusha, P. F. Harrison, N. M. Hartmann, Y. Hasegawa, A. Hasib, S. Hassani, S. Haug, R. Hauser, L. Hauswald, L. B. Havener, M. Havranek, C. M. Hawkes, R. J. Hawkings, D. Hayakawa, D. Hayden, C. P. Hays, J. M. Hays, H. S. Hayward, S. J. Haywood, S. J. Head, T. Heck, V. Hedberg, L. Heelan, S. Heer, K. K. Heidegger, S. Heim, T. Heim, B. Heinemann, J. J. Heinrich, L. Heinrich, C. Heinz, J. Hejbal, L. Helary, A. Held, S. Hellman, C. Helsens, R. C. W. Henderson, Y. Heng, S. Henkelmann, A. M. Henriques Correia, S. Henrot-Versille, G. H. Herbert, H. Herde, V. Herget, Y. Hernández Jiménez, H. Herr, G. Herten, R. Hertenberger, L. Hervas, T. C. Herwig, G. G. Hesketh, N. P. Hessey, J. W. Hetherly, S. Higashino, E. Higón-Rodriguez, K. Hildebrand, E. Hill, J. C. Hill, K. H. Hiller, S. J. Hillier, M. Hils, I. Hinchliffe, M. Hirose, D. Hirschbuehl, B. Hiti, O. Hladik, X. Hoad, J. Hobbs, N. Hod, M. C. Hodgkinson, P. Hodgson, A. Hoecker, M. R. Hoeferkamp, F. Hoenig, D. Hohn, T. R. Holmes, M. Homann, S. Honda, T. Honda, T. M. Hong, B. H. Hooberman, W. H. Hopkins, Y. Horii, A. J. Horton, J-Y. Hostachy, A. Hostiuc, S. Hou, A. Hoummada, J. Howarth, J. Hoya, M. Hrabovsky, J. Hrdinka, I. Hristova, J. Hrivnac, T. Hryn’ova, A. Hrynevich, P. J. Hsu, S.-C. Hsu, Q. Hu, S. Hu, Y. Huang, Z. Hubacek, F. Hubaut, F. Huegging, T. B. Huffman, E. W. Hughes, G. Hughes, M. Huhtinen, P. Huo, N. Huseynov, J. Huston, J. Huth, G. Iacobucci, G. Iakovidis, I. Ibragimov, L. Iconomidou-Fayard, Z. Idrissi, P. Iengo, O. Igonkina, T. Iizawa, Y. Ikegami, M. Ikeno, Y. Ilchenko, D. Iliadis, N. Ilic, G. Introzzi, P. Ioannou, M. Iodice, K. Iordanidou, V. Ippolito, M. F. Isacson, N. Ishijima, M. Ishino, M. Ishitsuka, C. Issever, S. Istin, F. Ito, J. M. Iturbe Ponce, R. Iuppa, H. Iwasaki, J. M. Izen, V. Izzo, S. Jabbar, P. Jackson, R. M. Jacobs, V. Jain, K. B. Jakobi, K. Jakobs, S. Jakobsen, T. Jakoubek, D. O. Jamin, D. K. Jana, R. Jansky, J. Janssen, M. Janus, P. A. Janus, G. Jarlskog, N. Javadov, T. Javůrek, M. Javurkova, F. Jeanneau, L. Jeanty, J. Jejelava, A. Jelinskas, P. Jenni, C. Jeske, S. Jézéquel, H. Ji, J. Jia, H. Jiang, Y. Jiang, Z. Jiang, S. Jiggins, J. Jimenez Pena, S. Jin, A. Jinaru, O. Jinnouchi, H. Jivan, P. Johansson, K. A. Johns, C. A. Johnson, W. J. Johnson, K. Jon-And, R. W. L. Jones, S. D. Jones, S. Jones, T. J. Jones, J. Jongmanns, P. M. Jorge, J. Jovicevic, X. Ju, A. Juste Rozas, M. K. Köhler, A. Kaczmarska, M. Kado, H. Kagan, M. Kagan, S. J. Kahn, T. Kaji, E. Kajomovitz, C. W. Kalderon, A. Kaluza, S. Kama, A. Kamenshchikov, N. Kanaya, L. Kanjir, V. A. Kantserov, J. Kanzaki, B. Kaplan, L. S. Kaplan, D. Kar, K. Karakostas, N. Karastathis, M. J. Kareem, E. Karentzos, S. N. Karpov, Z. M. Karpova, K. Karthik, V. Kartvelishvili, A. N. Karyukhin, K. Kasahara, L. Kashif, R. D. Kass, A. Kastanas, Y. Kataoka, C. Kato, A. Katre, J. Katzy, K. Kawade, K. Kawagoe, T. Kawamoto, G. Kawamura, E. F. Kay, V. F. Kazanin, R. Keeler, R. Kehoe, J. S. Keller, E. Kellermann, J. J. Kempster, J Kendrick, H. Keoshkerian, O. Kepka, B. P. Kerševan, S. Kersten, R. A. Keyes, M. Khader, F. Khalil-zada, A. Khanov, A. G. Kharlamov, T. Kharlamova, A. Khodinov, T. J. Khoo, V. Khovanskiy, E. Khramov, J. Khubua, S. Kido, C. R. Kilby, H. Y. Kim, S. H. Kim, Y. K. Kim, N. Kimura, O. M. Kind, B. T. King, D. Kirchmeier, J. Kirk, A. E. Kiryunin, T. Kishimoto, D. Kisielewska, V. Kitali, O. Kivernyk, E. Kladiva, T. Klapdor-Kleingrothaus, M. H. Klein, M. Klein, U. Klein, K. Kleinknecht, P. Klimek, A. Klimentov, R. Klingenberg, T. Klingl, T. Klioutchnikova, E.-E. Kluge, P. Kluit, S. Kluth, E. Kneringer, E. B. F. G. Knoops, A. Knue, A. Kobayashi, D. Kobayashi, T. Kobayashi, M. Kobel, M. Kocian, P. Kodys, T. Koffas, E. Koffeman, N. M. Köhler, T. Koi, M. Kolb, I. Koletsou, A. A. Komar, T. Kondo, N. Kondrashova, K. Köneke, A. C. König, T. Kono, R. Konoplich, N. Konstantinidis, R. Kopeliansky, S. Koperny, A. K. Kopp, K. Korcyl, K. Kordas, A. Korn, A. A. Korol, I. Korolkov, E. V. Korolkova, O. Kortner, S. Kortner, T. Kosek, V. V. Kostyukhin, A. Kotwal, A. Koulouris, A. Kourkoumeli-Charalampidi, C. Kourkoumelis, E. Kourlitis, V. Kouskoura, A. B. Kowalewska, R. Kowalewski, T. Z. Kowalski, C. Kozakai, W. Kozanecki, A. S. Kozhin, V. A. Kramarenko, G. Kramberger, D. Krasnopevtsev, M. W. Krasny, A. Krasznahorkay, D. Krauss, J. A. Kremer, J. Kretzschmar, K. Kreutzfeldt, P. Krieger, K. Krizka, K. Kroeninger, H. Kroha, J. Kroll, J. Kroll, J. Kroseberg, J. Krstic, U. Kruchonak, H. Krüger, N. Krumnack, M. C. Kruse, T. Kubota, H. Kucuk, S. Kuday, J. T. Kuechler, S. Kuehn, A. Kugel, F. Kuger, T. Kuhl, V. Kukhtin, R. Kukla, Y. Kulchitsky, S. Kuleshov, Y. P. Kulinich, M. Kuna, T. Kunigo, A. Kupco, T. Kupfer, O. Kuprash, H. Kurashige, L. L. Kurchaninov, Y. A. Kurochkin, M. G. Kurth, V. Kus, E. S. Kuwertz, M. Kuze, J. Kvita, T. Kwan, D. Kyriazopoulos, A. La Rosa, J. L. LaRosa Navarro, L. La Rotonda, F. La Ruffa, C. Lacasta, F. Lacava, J. Lacey, D. P. J. Lack, H. Lacker, D. Lacour, E. Ladygin, R. Lafaye, B. Laforge, T. Lagouri, S. Lai, S. Lammers, W. Lampl, E. Lançon, U. Landgraf, M. P. J. Landon, M. C. Lanfermann, V. S. Lang, J. C. Lange, R. J. Langenberg, A. J. Lankford, F. Lanni, K. Lantzsch, A. Lanza, A. Lapertosa, S. Laplace, J. F. Laporte, T. Lari, F. Lasagni Manghi, M. Lassnig, T. S. Lau, P. Laurelli, W. Lavrijsen, A. T. Law, P. Laycock, T. Lazovich, M. Lazzaroni, B. Le, O. Le Dortz, E. Le Guirriec, E. P. Le Quilleuc, M. LeBlanc, T. Le Compte, F. Ledroit-Guillon, C. A. Lee, G. R. Lee, S. C. Lee, L. Lee, B. Lefebvre, G. Lefebvre, M. Lefebvre, F. Legger, C. Leggett, G. Lehmann Miotto, X. Lei, W. A. Leight, M. A. L. Leite, R. Leitner, D. Lellouch, B. Lemmer, K. J. C. Leney, T. Lenz, B. Lenzi, R. Leone, S. Leone, C. Leonidopoulos, G. Lerner, C. Leroy, A. A. J. Lesage, C. G. Lester, M. Levchenko, J. Levêque, D. Levin, L. J. Levinson, M. Levy, D. Lewis, B. Li, Changqiao Li, H. Li, L. Li, Q. Li, Q. Li, S. Li, X. Li, Y. Li, Z. Liang, B. Liberti, A. Liblong, K. Lie, J. Liebal, W. Liebig, A. Limosani, S. C. Lin, T. H. Lin, R. A. Linck, B. E. Lindquist, A. E. Lionti, E. Lipeles, A. Lipniacka, M. Lisovyi, T. M. Liss, A. Lister, A. M. Litke, B. Liu, H. Liu, H. Liu, J. K. K. Liu, J. Liu, J. B. Liu, K. Liu, L. Liu, M. Liu, Y. L. Liu, Y. Liu, M. Livan, A. Lleres, J. Llorente Merino, S. L. Lloyd, C. Y. Lo, F. Lo Sterzo, E. M. Lobodzinska, P. Loch, F. K. Loebinger, A. Loesle, K. M. Loew, A. Loginov, T. Lohse, K. Lohwasser, M. Lokajicek, B. A. Long, J. D. Long, R. E. Long, L. Longo, K. A. Looper, J. A. Lopez, D. Lopez Mateos, I. Lopez Paz, A. Lopez Solis, J. Lorenz, N. Lorenzo Martinez, M. Losada, P. J. Lösel, X. Lou, A. Lounis, J. Love, P. A. Love, H. Lu, N. Lu, Y. J. Lu, H. J. Lubatti, C. Luci, A. Lucotte, C. Luedtke, F. Luehring, W. Lukas, L. Luminari, O. Lundberg, B. Lund-Jensen, M. S. Lutz, P. M. Luzi, D. Lynn, R. Lysak, E. Lytken, F. Lyu, V. Lyubushkin, H. Ma, L. L. Ma, Y. Ma, G. Maccarrone, A. Macchiolo, C. M. Macdonald, B. Maček, J. Machado Miguens, D. Madaffari, R. Madar, W. F. Mader, A. Madsen, J. Maeda, S. Maeland, T. Maeno, A. S. Maevskiy, V. Magerl, J. Mahlstedt, C. Maiani, C. Maidantchik, A. A. Maier, T. Maier, A. Maio, O. Majersky, S. Majewski, Y. Makida, N. Makovec, B. Malaescu, Pa. Malecki, V. P. Maleev, F. Malek, U. Mallik, D. Malon, C. Malone, S. Maltezos, S. Malyukov, J. Mamuzic, G. Mancini, I. Mandić, J. Maneira, L. Manhaes de Andrade Filho, J. Manjarres Ramos, K. H. Mankinen, A. Mann, A. Manousos, B. Mansoulie, J. D. Mansour, R. Mantifel, M. Mantoani, S. Manzoni, L. Mapelli, G. Marceca, L. March, L. Marchese, G. Marchiori, M. Marcisovsky, M. Marjanovic, D. E. Marley, F. Marroquim, S. P. Marsden, Z. Marshall, M. U. F Martensson, S. Marti-Garcia, C. B. Martin, T. A. Martin, V. J. Martin, B. Martin dit Latour, M. Martinez, V. I. Martinez Outschoorn, S. Martin-Haugh, V. S. Martoiu, A. C. Martyniuk, A. Marzin, L. Masetti, T. Mashimo, R. Mashinistov, J. Masik, A. L. Maslennikov, L. Massa, P. Mastrandrea, A. Mastroberardino, T. Masubuchi, P. Mättig, J. Maurer, S. J. Maxfield, D. A. Maximov, R. Mazini, I. Maznas, S. M. Mazza, N. C. Mc Fadden, G. Mc Goldrick, S. P. Mc Kee, A. McCarn, R. L. McCarthy, T. G. McCarthy, L. I. McClymont, E. F. McDonald, J. A. Mcfayden, G. Mchedlidze, S. J. Mc Mahon, P. C. McNamara, C. J. McNicol, R. A. McPherson, S. Meehan, T. J. Megy, S. Mehlhase, A. Mehta, T. Meideck, K. Meier, B. Meirose, D. Melini, B. R. Mellado Garcia, J. D. Mellenthin, M. Melo, F. Meloni, A. Melzer, S. B. Menary, L. Meng, X. T. Meng, A. Mengarelli, S. Menke, E. Meoni, S. Mergelmeyer, C. Merlassino, P. Mermod, L. Merola, C. Meroni, F. S. Merritt, A. Messina, J. Metcalfe, A. S. Mete, C. Meyer, J-P. Meyer, J. Meyer, H. Meyer Zu Theenhausen, F. Miano, R. P. Middleton, S. Miglioranzi, L. Mijović, G. Mikenberg, M. Mikestikova, M. Mikuž, M. Milesi, A. Milic, D. A. Millar, D. W. Miller, C. Mills, A. Milov, D. A. Milstead, A. A. Minaenko, Y. Minami, I. A. Minashvili, A. I. Mincer, B. Mindur, M. Mineev, Y. Minegishi, Y. Ming, L. M. Mir, K. P. Mistry, T. Mitani, J. Mitrevski, V. A. Mitsou, A. Miucci, P. S. Miyagawa, A. Mizukami, J. U. Mjörnmark, T. Mkrtchyan, M. Mlynarikova, T. Moa, K. Mochizuki, P. Mogg, S. Mohapatra, S. Molander, R. Moles-Valls, M. C. Mondragon, K. Mönig, J. Monk, E. Monnier, A. Montalbano, J. Montejo Berlingen, F. Monticelli, S. Monzani, R. W. Moore, N. Morange, D. Moreno, M. Moreno Llácer, P. Morettini, S. Morgenstern, D. Mori, T. Mori, M. Morii, M. Morinaga, V. Morisbak, A. K. Morley, G. Mornacchi, J. D. Morris, L. Morvaj, P. Moschovakos, M. Mosidze, H. J. Moss, J. Moss, K. Motohashi, R. Mount, E. Mountricha, E. J. W. Moyse, S. Muanza, F. Mueller, J. Mueller, R. S. P. Mueller, D. Muenstermann, P. Mullen, G. A. Mullier, F. J. Munoz Sanchez, W. J. Murray, H. Musheghyan, M. Muškinja, A. G. Myagkov, M. Myska, B. P. Nachman, O. Nackenhorst, K. Nagai, R. Nagai, K. Nagano, Y. Nagasaka, K. Nagata, M. Nagel, E. Nagy, A. M. Nairz, Y. Nakahama, K. Nakamura, T. Nakamura, I. Nakano, R. F. Naranjo Garcia, R. Narayan, D. I. Narrias Villar, I. Naryshkin, T. Naumann, G. Navarro, R. Nayyar, H. A. Neal, P. Yu. Nechaeva, T. J. Neep, A. Negri, M. Negrini, S. Nektarijevic, C. Nellist, A. Nelson, M. E. Nelson, S. Nemecek, P. Nemethy, M. Nessi, M. S. Neubauer, M. Neumann, P. R. Newman, T. Y. Ng, T. Nguyen Manh, R. B. Nickerson, R. Nicolaidou, J. Nielsen, V. Nikolaenko, I. Nikolic-Audit, K. Nikolopoulos, J. K. Nilsen, P. Nilsson, Y. Ninomiya, A. Nisati, N. Nishu, R. Nisius, I. Nitsche, T. Nitta, T. Nobe, Y. Noguchi, M. Nomachi, I. Nomidis, M. A. Nomura, T. Nooney, M. Nordberg, N. Norjoharuddeen, O. Novgorodova, M. Nozaki, L. Nozka, K. Ntekas, E. Nurse, F. Nuti, K. O’connor, D. C. O’Neil, A. A. O’Rourke, V. O’Shea, F. G. Oakham, H. Oberlack, T. Obermann, J. Ocariz, A. Ochi, I. Ochoa, J. P. Ochoa-Ricoux, S. Oda, S. Odaka, A. Oh, S. H. Oh, C. C. Ohm, H. Ohman, H. Oide, H. Okawa, Y. Okumura, T. Okuyama, A. Olariu, L. F. Oleiro Seabra, S. A. Olivares Pino, D. Oliveira Damazio, A. Olszewski, J. Olszowska, A. Onofre, K. Onogi, P. U. E. Onyisi, H. Oppen, M. J. Oreglia, Y. Oren, D. Orestano, N. Orlando, R. S. Orr, B. Osculati, R. Ospanov, G. Oteroy Garzon, H. Otono, M. Ouchrif, F. Ould-Saada, A. Ouraou, K. P. Oussoren, Q. Ouyang, M. Owen, R. E. Owen, V. E. Ozcan, N. Ozturk, K. Pachal, A. Pacheco Pages, L. Pacheco Rodriguez, C. Padilla Aranda, S. Pagan Griso, M. Paganini, F. Paige, G. Palacino, S. Palazzo, S. Palestini, M. Palka, D. Pallin, E. St.Panagiotopoulou, I. Panagoulias, C. E. Pandini, J. G. Panduro Vazquez, P. Pani, S. Panitkin, D. Pantea, L. Paolozzi, Th. D. Papadopoulou, K. Papageorgiou, A. Paramonov, D. Paredes Hernandez, A. J. Parker, M. A. Parker, K. A. Parker, F. Parodi, J. A. Parsons, U. Parzefall, V. R. Pascuzzi, J. M. Pasner, E. Pasqualucci, S. Passaggio, Fr. Pastore, S. Pataraia, J. R. Pater, T. Pauly, B. Pearson, S. Pedraza Lopez, R. Pedro, S. V. Peleganchuk, O. Penc, C. Peng, H. Peng, J. Penwell, B. S. Peralva, M. M. Perego, D. V. Perepelitsa, F. Peri, L. Perini, H. Pernegger, S. Perrella, R. Peschke, V. D. Peshekhonov, K. Peters, R. F. Y. Peters, B. A. Petersen, T. C. Petersen, E. Petit, A. Petridis, C. Petridou, P. Petroff, E. Petrolo, M. Petrov, F. Petrucci, N. E. Pettersson, A. Peyaud, R. Pezoa, F. H. Phillips, P. W. Phillips, G. Piacquadio, E. Pianori, A. Picazio, E. Piccaro, M. A. Pickering, R. Piegaia, J. E. Pilcher, A. D. Pilkington, A. W. J. Pin, M. Pinamonti, J. L. Pinfold, H. Pirumov, M. Pitt, L. Plazak, M.-A. Pleier, V. Pleskot, E. Plotnikova, D. Pluth, P. Podberezko, R. Poettgen, R. Poggi, L. Poggioli, I. Pogrebnyak, D. Pohl, G. Polesello, A. Poley, A. Policicchio, R. Polifka, A. Polini, C. S. Pollard, V. Polychronakos, K. Pommès, D. Ponomarenko, L. Pontecorvo, G. A. Popeneciu, D. M. PortilloQuintero, S. Pospisil, K. Potamianos, I. N. Potrap, C. J. Potter, H. Potti, T. Poulsen, J. Poveda, M. E. Pozo Astigarraga, P. Pralavorio, A. Pranko, S. Prell, D. Price, M. Primavera, S. Prince, N. Proklova, K. Prokofiev, F. Prokoshin, S. Protopopescu, J. Proudfoot, M. Przybycien, A. Puri, P. Puzo, J. Qian, G. Qin, Y. Qin, A. Quadt, M. Queitsch-Maitland, D. Quilty, S. Raddum, V. Radeka, V. Radescu, S. K. Radhakrishnan, P. Radloff, P. Rados, F. Ragusa, G. Rahal, J. A. Raine, S. Rajagopalan, C. Rangel-Smith, T. Rashid, S. Raspopov, M. G. Ratti, D. M. Rauch, F. Rauscher, S. Rave, I. Ravinovich, J. H. Rawling, M. Raymond, A. L. Read, N. P. Readioff, M. Reale, D. M. Rebuzzi, A. Redelbach, G. Redlinger, R. Reece, R. G. Reed, K. Reeves, L. Rehnisch, J. Reichert, A. Reiss, C. Rembser, H. Ren, M. Rescigno, S. Resconi, E. D. Resseguie, S. Rettie, E. Reynolds, O. L. Rezanova, P. Reznicek, R. Rezvani, R. Richter, S. Richter, E. Richter-Was, O. Ricken, M. Ridel, P. Rieck, C. J. Riegel, J. Rieger, O. Rifki, M. Rijssenbeek, A. Rimoldi, M. Rimoldi, L. Rinaldi, G. Ripellino, B. Ristić, E. Ritsch, I. Riu, F. Rizatdinova, E. Rizvi, C. Rizzi, R. T. Roberts, S. H. Robertson, A. Robichaud-Veronneau, D. Robinson, J. E. M. Robinson, A. Robson, E. Rocco, C. Roda, Y. Rodina, S. Rodriguez Bosca, A. Rodriguez Perez, D. Rodriguez Rodriguez, S. Roe, C. S. Rogan, O. Røhne, J. Roloff, A. Romaniouk, M. Romano, S. M. Romano Saez, E. Romero Adam, N. Rompotis, M. Ronzani, L. Roos, S. Rosati, K. Rosbach, P. Rose, N.-A. Rosien, E. Rossi, L. P. Rossi, J. H. N. Rosten, R. Rosten, M. Rotaru, J. Rothberg, D. Rousseau, A. Rozanov, Y. Rozen, X. Ruan, F. Rubbo, F. Rühr, A. Ruiz-Martinez, Z. Rurikova, N. A. Rusakovich, H. L. Russell, J. P. Rutherfoord, N. Ruthmann, Y. F. Ryabov, M. Rybar, G. Rybkin, S. Ryu, A. Ryzhov, G. F. Rzehorz, A. F. Saavedra, G. Sabato, S. Sacerdoti, H.F-W. Sadrozinski, R. Sadykov, F. Safai Tehrani, P. Saha, M. Sahinsoy, M. Saimpert, M. Saito, T. Saito, H. Sakamoto, Y. Sakurai, G. Salamanna, J. E. Salazar Loyola, D. Salek, P. H. Sales De Bruin, D. Salihagic, A. Salnikov, J. Salt, D. Salvatore, F. Salvatore, A. Salvucci, A. Salzburger, D. Sammel, D. Sampsonidis, D. Sampsonidou, J. Sánchez, V. Sanchez Martinez, A. Sanchez Pineda, H. Sandaker, R. L. Sandbach, C. O. Sander, M. Sandhoff, C. Sandoval, D. P. C. Sankey, M. Sannino, Y. Sano, A. Sansoni, C. Santoni, H. Santos, I. Santoyo Castillo, A. Sapronov, J. G. Saraiva, B. Sarrazin, O. Sasaki, K. Sato, E. Sauvan, G. Savage, P. Savard, N. Savic, C. Sawyer, L. Sawyer, J. Saxon, C. Sbarra, A. Sbrizzi, T. Scanlon, D. A. Scannicchio, J. Schaarschmidt, P. Schacht, B. M. Schachtner, D. Schaefer, L. Schaefer, R. Schaefer, J. Schaeffer, S. Schaepe, S. Schaetzel, U. Schäfer, A. C. Schaffer, D. Schaile, R. D. Schamberger, V. A. Schegelsky, D. Scheirich, M. Schernau, C. Schiavi, S. Schier, L. K. Schildgen, C. Schillo, M. Schioppa, S. Schlenker, K. R. Schmidt-Sommerfeld, K. Schmieden, C. Schmitt, S. Schmitt, S. Schmitz, U. Schnoor, L. Schoeffel, A. Schoening, B. D. Schoenrock, E. Schopf, M. Schott, J. F. P. Schouwenberg, J. Schovancova, S. Schramm, N. Schuh, A. Schulte, M. J. Schultens, H.-C. Schultz-Coulon, H. Schulz, M. Schumacher, B. A. Schumm, Ph. Schune, A. Schwartzman, T. A. Schwarz, H. Schweiger, Ph. Schwemling, R. Schwienhorst, J. Schwindling, A. Sciandra, G. Sciolla, M. Scornajenghi, F. Scuri, F. Scutti, J. Searcy, P. Seema, S. C. Seidel, A. Seiden, J. M. Seixas, G. Sekhniaidze, K. Sekhon, S. J. Sekula, N. Semprini-Cesari, S. Senkin, C. Serfon, L. Serin, L. Serkin, M. Sessa, R. Seuster, H. Severini, T. Sfiligoj, F. Sforza, A. Sfyrla, E. Shabalina, N. W. Shaikh, L. Y. Shan, R. Shang, J. T. Shank, M. Shapiro, P. B. Shatalov, K. Shaw, S. M. Shaw, A. Shcherbakova, C. Y. Shehu, Y. Shen, N. Sherafati, P. Sherwood, L. Shi, S. Shimizu, C. O. Shimmin, M. Shimojima, I. P. J. Shipsey, S. Shirabe, M. Shiyakova, J. Shlomi, A. Shmeleva, D. Shoaleh Saadi, M. J. Shochet, S. Shojaii, D. R. Shope, S. Shrestha, E. Shulga, M. A. Shupe, P. Sicho, A. M. Sickles, P. E. Sidebo, E. Sideras Haddad, O. Sidiropoulou, A. Sidoti, F. Siegert, Dj. Sijacki, J. Silva, S. B. Silverstein, V. Simak, Lj. Simic, S. Simion, E. Simioni, B. Simmons, M. Simon, P. Sinervo, N. B. Sinev, M. Sioli, G. Siragusa, I. Siral, S. Yu. Sivoklokov, J. Sjölin, M. B. Skinner, P. Skubic, M. Slater, T. Slavicek, M. Slawinska, K. Sliwa, R. Slovak, V. Smakhtin, B. H. Smart, J. Smiesko, N. Smirnov, S. Yu. Smirnov, Y. Smirnov, L. N. Smirnova, O. Smirnova, J. W. Smith, M. N. K. Smith, R. W. Smith, M. Smizanska, K. Smolek, A. A. Snesarev, I. M. Snyder, S. Snyder, R. Sobie, F. Socher, A. Soffer, A. Søgaard, D. A. Soh, G. Sokhrannyi, C. A. Solans Sanchez, M. Solar, E. Yu. Soldatov, U. Soldevila, A. A. Solodkov, A. Soloshenko, O. V. Solovyanov, V. Solovyev, P. Sommer, H. Son, A. Sopczak, D. Sosa, C. L. Sotiropoulou, R. Soualah, A. M. Soukharev, D. South, B. C. Sowden, S. Spagnolo, M. Spalla, M. Spangenberg, F. Spanò, D. Sperlich, F. Spettel, T. M. Spieker, R. Spighi, G. Spigo, L. A. Spiller, M. Spousta, R. D. St.Denis, A. Stabile, R. Stamen, S. Stamm, E. Stanecka, R. W. Stanek, C. Stanescu, M. M. Stanitzki, B. S. Stapf, S. Stapnes, E. A. Starchenko, G. H. Stark, J. Stark, S. H Stark, P. Staroba, P. Starovoitov, S. Stärz, R. Staszewski, P. Steinberg, B. Stelzer, H. J. Stelzer, O. Stelzer-Chilton, H. Stenzel, G. A. Stewart, M. C. Stockton, M. Stoebe, G. Stoicea, P. Stolte, S. Stonjek, A. R. Stradling, A. Straessner, M. E. Stramaglia, J. Strandberg, S. Strandberg, M. Strauss, P. Strizenec, R. Ströhmer, D. M. Strom, R. Stroynowski, A. Strubig, S. A. Stucci, B. Stugu, N. A. Styles, D. Su, J. Su, S. Suchek, Y. Sugaya, M. Suk, V. V. Sulin, D M S. Sultan, S. Sultansoy, T. Sumida, S. Sun, X. Sun, K. Suruliz, C. J. E. Suster, M. R. Sutton, S. Suzuki, M. Svatos, M. Swiatlowski, S. P. Swift, I. Sykora, T. Sykora, D. Ta, K. Tackmann, J. Taenzer, A. Taffard, R. Tafirout, E. Tahirovic, N. Taiblum, H. Takai, R. Takashima, E. H. Takasugi, T. Takeshita, Y. Takubo, M. Talby, A. A. Talyshev, J. Tanaka, M. Tanaka, R. Tanaka, S. Tanaka, R. Tanioka, B. B. Tannenwald, S. Tapia Araya, S. Tapprogge, S. Tarem, G. F. Tartarelli, P. Tas, M. Tasevsky, T. Tashiro, E. Tassi, A. Tavares Delgado, Y. Tayalati, A. C. Taylor, A. J. Taylor, G. N. Taylor, P. T. E. Taylor, W. Taylor, P. Teixeira-Dias, D. Temple, H. Ten Kate, P. K. Teng, J. J. Teoh, F. Tepel, S. Terada, K. Terashi, J. Terron, S. Terzo, M. Testa, R. J. Teuscher, T. Theveneaux-Pelzer, F. Thiele, J. P. Thomas, J. Thomas-Wilsker, P. D. Thompson, A. S. Thompson, L. A. Thomsen, E. Thomson, M. J. Tibbetts, R. E. Ticse Torres, V. O. Tikhomirov, Yu. A. Tikhonov, S. Timoshenko, P. Tipton, S. Tisserant, K. Todome, S. Todorova-Nova, S. Todt, J. Tojo, S. Tokár, K. Tokushuku, E. Tolley, L. Tomlinson, M. Tomoto, L. Tompkins, K. Toms, B. Tong, P. Tornambe, E. Torrence, H. Torres, E. Torró Pastor, J. Toth, F. Touchard, D. R. Tovey, C. J. Treado, T. Trefzger, F. Tresoldi, A. Tricoli, I. M. Trigger, S. Trincaz-Duvoid, M. F. Tripiana, W. Trischuk, B. Trocmé, A. Trofymov, C. Troncon, M. Trottier-McDonald, M. Trovatelli, L. Truong, M. Trzebinski, A. Trzupek, K. W. Tsang, J. C-L. Tseng, P. V. Tsiareshka, G. Tsipolitis, N. Tsirintanis, S. Tsiskaridze, V. Tsiskaridze, E. G. Tskhadadze, K. M. Tsui, I. I. Tsukerman, V. Tsulaia, S. Tsuno, D. Tsybychev, Y. Tu, A. Tudorache, V. Tudorache, T. T. Tulbure, A. N. Tuna, S. A. Tupputi, S. Turchikhin, D. Turgeman, I. Turk Cakir, R. Turra, P. M. Tuts, G. Ucchielli, I. Ueda, M. Ughetto, F. Ukegawa, G. Unal, A. Undrus, G. Unel, F. C. Ungaro, Y. Unno, C. Unverdorben, J. Urban, P. Urquijo, P. Urrejola, G. Usai, J. Usui, L. Vacavant, V. Vacek, B. Vachon, K. O. H. Vadla, A. Vaidya, C. Valderanis, E. Valdes Santurio, M. Valente, S. Valentinetti, A. Valero, L. Valéry, S. Valkar, A. Vallier, J. A. Valls Ferrer, W. Van Den Wollenberg, H. van der Graaf, P. van Gemmeren, J. Van Nieuwkoop, I. van Vulpen, M. C. van Woerden, M. Vanadia, W. Vandelli, A. Vaniachine, P. Vankov, G. Vardanyan, R. Vari, E. W. Varnes, C. Varni, T. Varol, D. Varouchas, A. Vartapetian, K. E. Varvell, J. G. Vasquez, G. A. Vasquez, F. Vazeille, T. Vazquez Schroeder, J. Veatch, V. Veeraraghavan, L. M. Veloce, F. Veloso, S. Veneziano, A. Ventura, M. Venturi, N. Venturi, A. Venturini, V. Vercesi, M. Verducci, W. Verkerke, A. T. Vermeulen, J. C. Vermeulen, M. C. Vetterli, N. Viaux Maira, O. Viazlo, I. Vichou, T. Vickey, O. E. Vickey Boeriu, G. H. A. Viehhauser, S. Viel, L. Vigani, M. Villa, M. Villaplana Perez, E. Vilucchi, M. G. Vincter, V. B. Vinogradov, A. Vishwakarma, C. Vittori, I. Vivarelli, S. Vlachos, M. Vogel, P. Vokac, G. Volpi, H. von der Schmitt, E. von Toerne, V. Vorobel, K. Vorobev, M. Vos, R. Voss, J. H. Vossebeld, N. Vranjes, M. Vranjes Milosavljevic, V. Vrba, M. Vreeswijk, R. Vuillermet, I. Vukotic, P. Wagner, W. Wagner, J. Wagner-Kuhr, H. Wahlberg, S. Wahrmund, J. Walder, R. Walker, W. Walkowiak, V. Wallangen, C. Wang, C. Wang, F. Wang, H. Wang, H. Wang, J. Wang, J. Wang, Q. Wang, R. Wang, S. M. Wang, T. Wang, W. Wang, W. Wang, Z. Wang, C. Wanotayaroj, A. Warburton, C. P. Ward, D. R. Wardrope, A. Washbrook, P. M. Watkins, A. T. Watson, M. F. Watson, G. Watts, S. Watts, B. M. Waugh, A. F. Webb, S. Webb, M. S. Weber, S. W. Weber, S. A. Weber, J. S. Webster, A. R. Weidberg, B. Weinert, J. Weingarten, M. Weirich, C. Weiser, H. Weits, P. S. Wells, T. Wenaus, T. Wengler, S. Wenig, N. Wermes, M. D. Werner, P. Werner, M. Wessels, T. D. Weston, K. Whalen, N. L. Whallon, A. M. Wharton, A. S. White, A. White, M. J. White, R. White, D. Whiteson, B. W. Whitmore, F. J. Wickens, W. Wiedenmann, M. Wielers, C. Wiglesworth, L. A. M. Wiik-Fuchs, A. Wildauer, F. Wilk, H. G. Wilkens, H. H. Williams, S. Williams, C. Willis, S. Willocq, J. A. Wilson, I. Wingerter-Seez, E. Winkels, F. Winklmeier, O. J. Winston, B. T. Winter, M. Wittgen, M. Wobisch, T. M. H. Wolf, R. Wolff, M. W. Wolter, H. Wolters, V. W. S. Wong, S. D. Worm, B. K. Wosiek, J. Wotschack, K. W. Wozniak, M. Wu, S. L. Wu, X. Wu, Y. Wu, T. R. Wyatt, B. M. Wynne, S. Xella, Z. Xi, L. Xia, D. Xu, L. Xu, T. Xu, B. Yabsley, S. Yacoob, D. Yamaguchi, Y. Yamaguchi, A. Yamamoto, S. Yamamoto, T. Yamanaka, F. Yamane, M. Yamatani, Y. Yamazaki, Z. Yan, H. Yang, H. Yang, Y. Yang, Z. Yang, W-M. Yao, Y. C. Yap, Y. Yasu, E. Yatsenko, K. H. YauWong, J. Ye, S. Ye, I. Yeletskikh, E. Yigitbasi, E. Yildirim, K. Yorita, K. Yoshihara, C. Young, C. J. S. Young, J. Yu, J. Yu, S. P. Y. Yuen, I. Yusuff, B. Zabinski, G. Zacharis, R. Zaidan, A. M. Zaitsev, N. Zakharchuk, J. Zalieckas, A. Zaman, S. Zambito, D. Zanzi, C. Zeitnitz, G. Zemaityte, A. Zemla, J. C. Zeng, Q. Zeng, O. Zenin, T. Ženiš, D. Zerwas, D. Zhang, F. Zhang, G. Zhang, H. Zhang, J. Zhang, L. Zhang, L. Zhang, M. Zhang, P. Zhang, R. Zhang, R. Zhang, X. Zhang, Y. Zhang, Z. Zhang, X. Zhao, Y. Zhao, Z. Zhao, A. Zhemchugov, B. Zhou, C. Zhou, L. Zhou, M. Zhou, M. Zhou, N. Zhou, C. G. Zhu, H. Zhu, J. Zhu, Y. Zhu, X. Zhuang, K. Zhukov, A. Zibell, D. Zieminska, N. I. Zimine, C. Zimmermann, S. Zimmermann, Z. Zinonos, M. Zinser, M. Ziolkowski, L. Živković, G. Zobernig, A. Zoccoli, R. Zou, M. zur Nedden, L. Zwalinski, ATLAS Collaboration

**Affiliations:** 1 Department of Physics, University of Adelaide, Adelaide, Australia; 2 Physics Department, SUNY Albany, Albany, NY USA; 3 Department of Physics, University of Alberta, Edmonton, AB Canada; 4 Department of Physics, Ankara University, Ankara, Turkey; 5 Istanbul Aydin University, Istanbul, Turkey; 6 Division of Physics, TOBB University of Economics and Technology, Ankara, Turkey; 7 LAPP, CNRS/IN2P3 and Université Savoie Mont Blanc, Annecy-le-Vieux, France; 8 High Energy Physics Division, Argonne National Laboratory, Argonne, IL USA; 9 Department of Physics, University of Arizona, Tucson, AZ USA; 10 Department of Physics, The University of Texas at Arlington, Arlington, TX USA; 11 Physics Department, National and Kapodistrian University of Athens, Athens, Greece; 12 Physics Department, National Technical University of Athens, Zografou, Greece; 13 Department of Physics, The University of Texas at Austin, Austin, TX USA; 14 Institute of Physics, Azerbaijan Academy of Sciences, Baku, Azerbaijan; 15 Institut de Física d’Altes Energies (IFAE), The Barcelona Institute of Science and Technology, Barcelona, Spain; 16 Institute of Physics, University of Belgrade, Belgrade, Serbia; 17 Department for Physics and Technology, University of Bergen, Bergen, Norway; 18 Physics Division, Lawrence Berkeley National Laboratory, University of California, Berkeley, CA USA; 19 Department of Physics, Humboldt University, Berlin, Germany; 20 Albert Einstein Center for Fundamental Physics, Laboratory for High Energy Physics, University of Bern, Bern, Switzerland; 21 School of Physics and Astronomy, University of Birmingham, Birmingham, UK; 22 Department of Physics, Bogazici University, Istanbul, Turkey; 23 Department of Physics Engineering, Gaziantep University, Gaziantep, Turkey; 24 Faculty of Engineering and Natural Sciences, Istanbul Bilgi University, Istanbul, Turkey; 25 Faculty of Engineering and Natural Sciences, Bahcesehir University, Istanbul, Turkey; 26 Centro de Investigaciones, Universidad Antonio Narino, Bogotá, Colombia; 27 INFN Sezione di Bologna, Bologna, Italy; 28 Dipartimento di Fisica e Astronomia, Università di Bologna, Bologna, Italy; 29 Physikalisches Institut, University of Bonn, Bonn, Germany; 30 Department of Physics, Boston University, Boston, MA USA; 31 Department of Physics, Brandeis University, Waltham, MA USA; 32 Universidade Federal do Rio De Janeiro COPPE/EE/IF, Rio de Janeiro, Brazil; 33 Electrical Circuits Department, Federal University of Juiz de Fora (UFJF), Juiz de Fora, Brazil; 34 Federal University of Sao Joao del Rei (UFSJ), Sao Joao del Rei, Brazil; 35 Instituto de Fisica, Universidade de Sao Paulo, São Paulo, Brazil; 36 Physics Department, Brookhaven National Laboratory, Upton, NY USA; 37 Transilvania University of Brasov, Brasov, Romania; 38 Horia Hulubei National Institute of Physics and Nuclear Engineering, Bucharest, Romania; 39 Department of Physics, Alexandru Ioan Cuza University of Iasi, Iasi, Romania; 40 Physics Department, National Institute for Research and Development of Isotopic and Molecular Technologies, Cluj-Napoca, Romania; 41 University Politehnica Bucharest, Bucharest, Romania; 42 West University in Timisoara, Timisoara, Romania; 43 Departamento de Física, Universidad de Buenos Aires, Buenos Aires, Argentina; 44 Cavendish Laboratory, University of Cambridge, Cambridge, UK; 45 Department of Physics, Carleton University, Ottawa, ON Canada; 46 CERN, Geneva, Switzerland; 47 Enrico Fermi Institute, University of Chicago, Chicago, IL USA; 48 Departamento de Física, Pontificia Universidad Católica de Chile, Santiago, Chile; 49 Departamento de Física, Universidad Técnica Federico Santa María, Valparaiso, Chile; 50 Institute of High Energy Physics, Chinese Academy of Sciences, Beijing, China; 51 Department of Physics, Nanjing University, Nanjing, Jiangsu China; 52 Physics Department, Tsinghua University, Beijing, 100084 China; 53 Department of Modern Physics and State Key Laboratory of Particle Detection and Electronics, University of Science and Technology of China, Hefei, Anhui China; 54 School of Physics, Shandong University, Jinan, Shandong China; 55 Department of Physics and Astronomy, Key Laboratory for Particle Physics, Astrophysics and Cosmology, Ministry of Education, Shanghai Key Laboratory for Particle Physics and Cosmology, Shanghai Jiao Tong University, Shanghai (also at PKU-CHEP), Shanghai, China; 56 Université Clermont Auvergne, CNRS/IN2P3, LPC, Clermont-Ferrand, France; 57 Nevis Laboratory, Columbia University, Irvington, NY USA; 58 Niels Bohr Institute, University of Copenhagen, Copenhagen, Denmark; 59 INFN Gruppo Collegato di Cosenza, Laboratori Nazionali di Frascati, Frascati, Italy; 60 Dipartimento di Fisica, Università della Calabria, Rende, Italy; 61 Faculty of Physics and Applied Computer Science, AGH University of Science and Technology, Kraków, Poland; 62 Marian Smoluchowski Institute of Physics, Jagiellonian University, Kraków, Poland; 63 Institute of Nuclear Physics, Polish Academy of Sciences, Kraków, Poland; 64 Physics Department, Southern Methodist University, Dallas, TX USA; 65 Physics Department, University of Texas at Dallas, Richardson, TX USA; 66 DESY, Hamburg and Zeuthen, Germany; 67 Lehrstuhl für Experimentelle Physik IV, Technische Universität Dortmund, Dortmund, Germany; 68 Institut für Kern- und Teilchenphysik, Technische Universität Dresden, Dresden, Germany; 69 Department of Physics, Duke University, Durham, NC USA; 70 SUPA-School of Physics and Astronomy, University of Edinburgh, Edinburgh, UK; 71 INFN e Laboratori Nazionali di Frascati, Frascati, Italy; 72 Fakultät für Mathematik und Physik, Albert-Ludwigs-Universität, Freiburg, Germany; 73 Departement de Physique Nucleaire et Corpusculaire, Université de Genève, Geneva, Switzerland; 74 INFN Sezione di Genova, Genoa, Italy; 75 Dipartimento di Fisica, Università di Genova, Genoa, Italy; 76 E. Andronikashvili Institute of Physics, Iv. Javakhishvili Tbilisi State University, Tbilisi, Georgia; 77 High Energy Physics Institute, Tbilisi State University, Tbilisi, Georgia; 78 II Physikalisches Institut, Justus-Liebig-Universität Giessen, Giessen, Germany; 79 SUPA-School of Physics and Astronomy, University of Glasgow, Glasgow, UK; 80 II Physikalisches Institut, Georg-August-Universität, Göttingen, Germany; 81 Laboratoire de Physique Subatomique et de Cosmologie, Université Grenoble-Alpes, CNRS/IN2P3, Grenoble, France; 82 Laboratory for Particle Physics and Cosmology, Harvard University, Cambridge, MA USA; 83 Kirchhoff-Institut für Physik, Ruprecht-Karls-Universität Heidelberg, Heidelberg, Germany; 84 Physikalisches Institut, Ruprecht-Karls-Universität Heidelberg, Heidelberg, Germany; 85 Faculty of Applied Information Science, Hiroshima Institute of Technology, Hiroshima, Japan; 86 Department of Physics, The Chinese University of Hong Kong, Shatin, NT Hong Kong; 87 Department of Physics, The University of Hong Kong, Hong Kong, China; 88 Department of Physics, Institute for Advanced Study, The Hong Kong University of Science and Technology, Clear Water Bay, Kowloon, Hong Kong, China; 89 Department of Physics, National Tsing Hua University, Taiwan, Taiwan; 90 Department of Physics, Indiana University, Bloomington, IN USA; 91 Institut für Astro- und Teilchenphysik, Leopold-Franzens-Universität, Innsbruck, Austria; 92 University of Iowa, Iowa City, IA USA; 93 Department of Physics and Astronomy, Iowa State University, Ames, IA USA; 94 Joint Institute for Nuclear Research, JINR Dubna, Dubna, Russia; 95 KEK, High Energy Accelerator Research Organization, Tsukuba, Japan; 96 Graduate School of Science, Kobe University, Kobe, Japan; 97 Faculty of Science, Kyoto University, Kyoto, Japan; 98 Kyoto University of Education, Kyoto, Japan; 99 Research Center for Advanced Particle Physics and Department of Physics, Kyushu University, Fukuoka, Japan; 100 Instituto de Física La Plata, Universidad Nacional de La Plata and CONICET, La Plata, Argentina; 101 Physics Department, Lancaster University, Lancaster, UK; 102 INFN Sezione di Lecce, Lecce, Italy; 103 Dipartimento di Matematica e Fisica, Università del Salento, Lecce, Italy; 104 Oliver Lodge Laboratory, University of Liverpool, Liverpool, UK; 105 Department of Experimental Particle Physics, Jožef Stefan Institute and Department of Physics, University of Ljubljana, Ljubljana, Slovenia; 106 School of Physics and Astronomy, Queen Mary University of London, London, UK; 107 Department of Physics, Royal Holloway University of London, Surrey, UK; 108 Department of Physics and Astronomy, University College London, London, UK; 109 Louisiana Tech University, Ruston, LA USA; 110 Laboratoire de Physique Nucléaire et de Hautes Energies, UPMC and Université Paris-Diderot and CNRS/IN2P3, Paris, France; 111 Fysiska institutionen, Lunds universitet, Lund, Sweden; 112 Departamento de Fisica Teorica C-15, Universidad Autonoma de Madrid, Madrid, Spain; 113 Institut für Physik, Universität Mainz, Mainz, Germany; 114 School of Physics and Astronomy, University of Manchester, Manchester, UK; 115 CPPM, Aix-Marseille Université and CNRS/IN2P3, Marseille, France; 116 Department of Physics, University of Massachusetts, Amherst, MA USA; 117 Department of Physics, McGill University, Montreal, QC Canada; 118 School of Physics, University of Melbourne, Victoria, Australia; 119 Department of Physics, The University of Michigan, Ann Arbor, MI USA; 120 Department of Physics and Astronomy, Michigan State University, East Lansing, MI USA; 121 INFN Sezione di Milano, Milan, Italy; 122 Dipartimento di Fisica, Università di Milano, Milan, Italy; 123 B.I. Stepanov Institute of Physics, National Academy of Sciences of Belarus, Minsk, Republic of Belarus; 124 Research Institute for Nuclear Problems of Byelorussian State University, Minsk, Republic of Belarus; 125 Group of Particle Physics, University of Montreal, Montreal, QC Canada; 126 P.N. Lebedev Physical Institute of the Russian Academy of Sciences, Moscow, Russia; 127 Institute for Theoretical and Experimental Physics (ITEP), Moscow, Russia; 128 National Research Nuclear University MEPhI, Moscow, Russia; 129 D.V. Skobeltsyn Institute of Nuclear Physics, M.V. Lomonosov Moscow State University, Moscow, Russia; 130 Fakultät für Physik, Ludwig-Maximilians-Universität München, Munich, Germany; 131 Max-Planck-Institut für Physik (Werner-Heisenberg-Institut), Munich, Germany; 132 Nagasaki Institute of Applied Science, Nagasaki, Japan; 133 Graduate School of Science and Kobayashi-Maskawa Institute, Nagoya University, Nagoya, Japan; 134 INFN Sezione di Napoli, Naples, Italy; 135 Dipartimento di Fisica, Università di Napoli, Naples, Italy; 136 Department of Physics and Astronomy, University of New Mexico, Albuquerque, NM USA; 137 Institute for Mathematics, Astrophysics and Particle Physics, Radboud University Nijmegen/Nikhef, Nijmegen, The Netherlands; 138 Nikhef National Institute for Subatomic Physics, University of Amsterdam, Amsterdam, The Netherlands; 139 Department of Physics, Northern Illinois University, DeKalb, IL USA; 140 Budker Institute of Nuclear Physics, SB RAS, Novosibirsk, Russia; 141 Department of Physics, New York University, New York, NY USA; 142 Ohio State University, Columbus, OH USA; 143 Faculty of Science, Okayama University, Okayama, Japan; 144 Homer L. Dodge Department of Physics and Astronomy, University of Oklahoma, Norman, OK USA; 145 Department of Physics, Oklahoma State University, Stillwater, OK USA; 146 Palacký University, RCPTM, Olomouc, Czech Republic; 147 Center for High Energy Physics, University of Oregon, Eugene, OR USA; 148 LAL, Univ. Paris-Sud, CNRS/IN2P3, Université Paris-Saclay, Orsay, France; 149 Graduate School of Science, Osaka University, Osaka, Japan; 150 Department of Physics, University of Oslo, Oslo, Norway; 151 Department of Physics, Oxford University, Oxford, UK; 152 INFN Sezione di Pavia, Pavia, Italy; 153 Dipartimento di Fisica, Università di Pavia, Pavia, Italy; 154 Department of Physics, University of Pennsylvania, Philadelphia, PA USA; 155 National Research Centre “Kurchatov Institute” B.P. Konstantinov Petersburg Nuclear Physics Institute, St. Petersburg, Russia; 156 INFN Sezione di Pisa, Pisa, Italy; 157 Dipartimento di Fisica E. Fermi, Università di Pisa, Pisa, Italy; 158 Department of Physics and Astronomy, University of Pittsburgh, Pittsburgh, PA USA; 159 Laboratório de Instrumentação e Física Experimental de Partículas-LIP, Lisbon, Portugal; 160 Faculdade de Ciências, Universidade de Lisboa, Lisbon, Portugal; 161 Department of Physics, University of Coimbra, Coimbra, Portugal; 162 Centro de Física Nuclear da Universidade de Lisboa, Lisbon, Portugal; 163 Departamento de Fisica, Universidade do Minho, Braga, Portugal; 164 Departamento de Fisica Teorica y del Cosmos, Universidad de Granada, Granada, Spain; 165 Dep Fisica and CEFITEC of Faculdade de Ciencias e Tecnologia, Universidade Nova de Lisboa, Caparica, Portugal; 166 Institute of Physics, Academy of Sciences of the Czech Republic, Prague, Czech Republic; 167 Czech Technical University in Prague, Prague, Czech Republic; 168 Faculty of Mathematics and Physics, Charles University, Prague, Czech Republic; 169 State Research Center Institute for High Energy Physics (Protvino), NRC KI, Protvino, Russia; 170 Particle Physics Department, Rutherford Appleton Laboratory, Didcot, UK; 171 INFN Sezione di Roma, Rome, Italy; 172 Dipartimento di Fisica, Sapienza Università di Roma, Rome, Italy; 173 INFN Sezione di Roma Tor Vergata, Rome, Italy; 174 Dipartimento di Fisica, Università di Roma Tor Vergata, Rome, Italy; 175 INFN Sezione di Roma Tre, Rome, Italy; 176 Dipartimento di Matematica e Fisica, Università Roma Tre, Rome, Italy; 177 Faculté des Sciences Ain Chock, Réseau Universitaire de Physique des Hautes Energies-Université Hassan II, Casablanca, Morocco; 178 Centre National de l’Energie des Sciences Techniques Nucleaires, Rabat, Morocco; 179 Faculté des Sciences Semlalia, Université Cadi Ayyad, LPHEA-Marrakech, Marrakech, Morocco; 180 Faculté des Sciences, Université Mohamed Premier and LPTPM, Oujda, Morocco; 181 Faculté des Sciences, Université Mohammed V, Rabat, Morocco; 182 DSM/IRFU (Institut de Recherches sur les Lois Fondamentales de l’Univers), CEA Saclay (Commissariat à l’Energie Atomique et aux Energies Alternatives), Gif-sur-Yvette, France; 183 Santa Cruz Institute for Particle Physics, University of California Santa Cruz, Santa Cruz, CA USA; 184 Department of Physics, University of Washington, Seattle, WA USA; 185 Department of Physics and Astronomy, University of Sheffield, Sheffield, UK; 186 Department of Physics, Shinshu University, Nagano, Japan; 187 Department Physik, Universität Siegen, Siegen, Germany; 188 Department of Physics, Simon Fraser University, Burnaby, BC Canada; 189 SLAC National Accelerator Laboratory, Stanford, CA USA; 190 Faculty of Mathematics, Physics and Informatics, Comenius University, Bratislava, Slovak Republic; 191 Department of Subnuclear Physics, Institute of Experimental Physics of the Slovak Academy of Sciences, Kosice, Slovak Republic; 192 Department of Physics, University of Cape Town, Cape Town, South Africa; 193 Department of Physics, University of Johannesburg, Johannesburg, South Africa; 194 School of Physics, University of the Witwatersrand, Johannesburg, South Africa; 195 Department of Physics, Stockholm University, Stockholm, Sweden; 196 The Oskar Klein Centre, Stockholm, Sweden; 197 Physics Department, Royal Institute of Technology, Stockholm, Sweden; 198 Departments of Physics and Astronomy and Chemistry, Stony Brook University, Stony Brook, NY USA; 199 Department of Physics and Astronomy, University of Sussex, Brighton, UK; 200 School of Physics, University of Sydney, Sydney, Australia; 201 Institute of Physics, Academia Sinica, Taipei, Taiwan; 202 Department of Physics, Technion: Israel Institute of Technology, Haifa, Israel; 203 Raymond and Beverly Sackler School of Physics and Astronomy, Tel Aviv University, Tel Aviv, Israel; 204 Department of Physics, Aristotle University of Thessaloniki, Thessaloníki, Greece; 205 International Center for Elementary Particle Physics and Department of Physics, The University of Tokyo, Tokyo, Japan; 206 Graduate School of Science and Technology, Tokyo Metropolitan University, Tokyo, Japan; 207 Department of Physics, Tokyo Institute of Technology, Tokyo, Japan; 208 Tomsk State University, Tomsk, Russia; 209 Department of Physics, University of Toronto, Toronto, ON Canada; 210 INFN-TIFPA, Trento, Italy; 211 University of Trento, Trento, Italy; 212 TRIUMF, Vancouver, BC Canada; 213 Department of Physics and Astronomy, York University, Toronto, ON Canada; 214 Faculty of Pure and Applied Sciences, and Center for Integrated Research in Fundamental Science and Engineering, University of Tsukuba, Tsukuba, Japan; 215 Department of Physics and Astronomy, Tufts University, Medford, MA USA; 216 Department of Physics and Astronomy, University of California Irvine, Irvine, CA USA; 217 INFN Gruppo Collegato di Udine, Sezione di Trieste, Udine, Italy; 218 ICTP, Trieste, Italy; 219 Dipartimento di Chimica, Fisica e Ambiente, Università di Udine, Udine, Italy; 220 Department of Physics and Astronomy, University of Uppsala, Uppsala, Sweden; 221 Department of Physics, University of Illinois, Urbana, IL USA; 222 Instituto de Fisica Corpuscular (IFIC), Centro Mixto Universidad de Valencia - CSIC, Valencia, Spain; 223 Department of Physics, University of British Columbia, Vancouver, BC Canada; 224 Department of Physics and Astronomy, University of Victoria, Victoria, BC Canada; 225 Department of Physics, University of Warwick, Coventry, UK; 226 Waseda University, Tokyo, Japan; 227 Department of Particle Physics, The Weizmann Institute of Science, Rehovot, Israel; 228 Department of Physics, University of Wisconsin, Madison, WI USA; 229 Fakultät für Physik und Astronomie, Julius-Maximilians-Universität, Würzburg, Germany; 230 Fakultät für Mathematik und Naturwissenschaften, Fachgruppe Physik, Bergische Universität Wuppertal, Wuppertal, Germany; 231 Department of Physics, Yale University, New Haven, CT USA; 232 Yerevan Physics Institute, Yerevan, Armenia; 233 Centre de Calcul de l’Institut National de Physique Nucléaire et de Physique des Particules (IN2P3), Villeurbanne, France; 234 Academia Sinica Grid Computing, Institute of Physics, Academia Sinica, Taipei, Taiwan; 235 CERN, 1211 Geneva 23, Switzerland

## Abstract

The results of a search for direct pair production of top squarks in events with two opposite-charge leptons (electrons or muons) are reported, using $36.1~\hbox {fb}^{-1}$ of integrated luminosity from proton–proton collisions at $\sqrt{s}=13$ TeV collected by the ATLAS detector at the Large Hadron Collider. To cover a range of mass differences between the top squark $\tilde{t}$ and lighter supersymmetric particles, four possible decay modes of the top squark are targeted with dedicated selections: the decay $\tilde{t} \rightarrow b \tilde{\chi }_{1}^{\pm }$ into a *b*-quark and the lightest chargino with $\tilde{\chi }_{1}^{\pm } \rightarrow W \tilde{\chi }_{1}^{0}$, the decay $\tilde{t} \rightarrow t \tilde{\chi }_{1}^{0}$ into an on-shell top quark and the lightest neutralino, the three-body decay $\tilde{t} \rightarrow b W \tilde{\chi }_{1}^{0}$ and the four-body decay $\tilde{t} \rightarrow b \ell \nu \tilde{\chi }_{1}^{0}$. No significant excess of events is observed above the Standard Model background for any selection, and limits on top squarks are set as a function of the $\tilde{t}$ and $\tilde{\chi }_{1}^{0}$ masses. The results exclude at 95% confidence level $\tilde{t}$ masses up to about 720 GeV, extending the exclusion region of supersymmetric parameter space covered by previous searches.

## Introduction

The standard model (SM) of particle physics is extremely successful in describing the phenomena of elementary particles and their interactions. Nevertheless, it is believed to be only a low-energy realisation of a more general theory. In its current form, it fails to explain several observations, such as the nature of dark matter, the baryon asymmetry of the universe and the stabilisation of the Higgs boson mass against radiative corrections from the Planck scale. These shortcomings could be remedied by the existence of new particles at the TeV scale, which motivates extensive searches at the Large Hadron Collider (LHC).

One of the most compelling theories beyond the SM is Supersymmetry (SUSY) [1–6]. SUSY is a space-time symmetry that for each SM particle postulates the existence of a partner particle whose spin (*S*) differs by one-half unit. The introduction of gauge-invariant and renormalisable interactions into SUSY models can violate the conservation of baryon number (*B*) and lepton number (*L*), resulting in a proton lifetime shorter than current experimental limits [7]. This is usually solved by assuming that the multiplicative quantum number *R*-parity [8], defined as $R~=~(-1)^{3(B-L)+2S}$, is conserved.

In the framework of a generic *R*-parity-conserving model, SUSY particles are produced in pairs, and the lightest supersymmetric particle (LSP) is stable and a candidate for dark matter [9, 10]. The scalar partners of right-handed and left-handed quarks (squarks), $\tilde{q}_{\mathrm {R}}$ and $\tilde{q}_{\mathrm {L}}$, can mix to form two mass eigenstates, $\tilde{q}_1$ and $\tilde{q}_2$, with $\tilde{q}_1$ defined to be the lighter one. In the case of the supersymmetric partner of the top quark, $\tilde{t}$, large mixing effects can lead to one top squark mass eigenstate, $\tilde{t}_1$, that is significantly lighter than the other squarks. The charginos and neutralinos are mixtures of the bino, winos and Higgsinos that are superpartners of the U(1) and SU(2) gauge bosons and the Higgs bosons, respectively. Their mass eigenstates are referred to as $\tilde{\chi }_{i}^{\pm }$ $(i=1,2)$ and $\tilde{\chi }_{j}^{0}$ $(j=1,2,3,4)$ in order of increasing masses. In a large variety of models, the LSP is the lightest neutralino $\tilde{\chi }^0_1$.

**Fig. 1:**
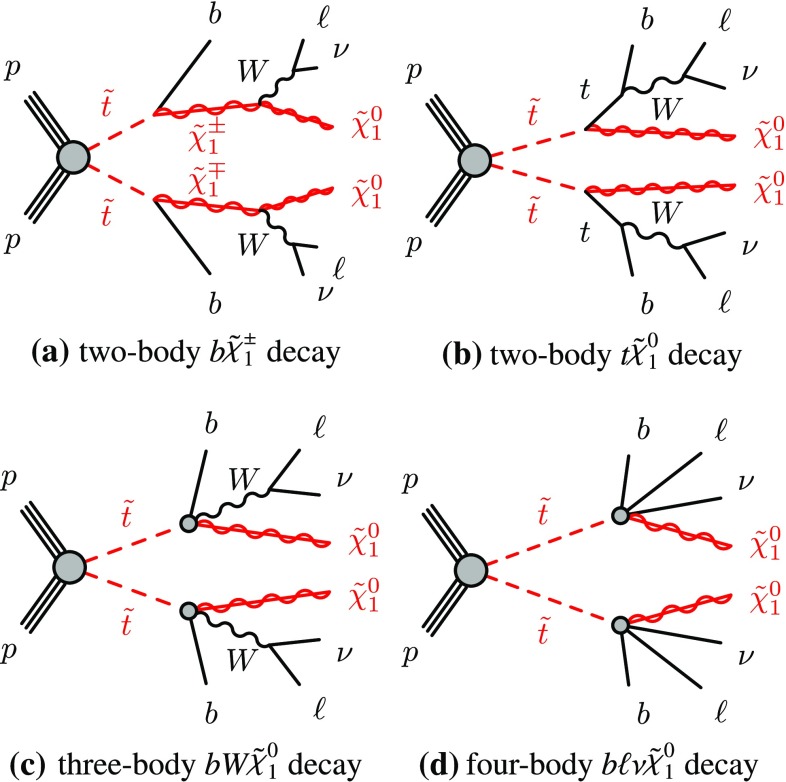
Diagrams representing the four main signals targeted by the analyses: **a** the decay of the top squark via the lightest chargino ($\tilde{t} \rightarrow b {\tilde{\chi }^\pm _1}$), **b** the two-body decay into an on-shell top quark and the lightest neutralino ($\tilde{t} \rightarrow t {\tilde{\chi }^0_1}$), **c** the three-body decay mode into an on-shell *W* boson, a *b*-quark and the lightest neutralino ($\tilde{t} \rightarrow b W {\tilde{\chi }^0_1}$) and **d** the four-body decay mode ($\tilde{t} \rightarrow b f f' {\tilde{\chi }^0_1}$) where the two fermions *f* and $f'$ are a lepton with its neutrino in this article

In this paper a search for direct pair production of the top squark is reported, in final states with two isolated leptons (electrons or muons) and missing transverse momentum. The search utilises $36.1~\hbox {fb}^{-1}$ of proton–proton collision data collected by the ATLAS experiment in 2015 and 2016 at a centre-of-mass energy $\sqrt{s}=13$ TeV.

The top squark is assumed to decay into either the lightest chargino or the lightest neutralino. Depending on the mass difference between the top squark and the lighter SUSY particles, different decay modes are relevant. The decays $\tilde{t} \rightarrow t {\tilde{\chi }^0_1}$ and $\tilde{t} \rightarrow b {\tilde{\chi }^\pm _1}$ (where *t* and *b* represent either the quark or the anti-quark, depending on the charge conjugation) with ${\tilde{\chi }^\pm _1} \rightarrow W {\tilde{\chi }^0_1}$ dominate when they are kinematically accessible. For intermediate mass differences, $m_{{\tilde{\chi }^0_1}}+m_{W}+m_{b}< m_{\tilde{t}} < m_{{\tilde{\chi }^0_1}}+m_{t}$, the three-body decay $\tilde{t} \rightarrow b W {\tilde{\chi }^0_1}$ is considered. For smaller mass differences, the four-body decay channel $\tilde{t} \rightarrow b f f' {\tilde{\chi }^0_1}$, where *f* and $f'$ are two fermions from the $W^{*}$ decay, is assumed to occur. In this search, *f* and $f'$ are a lepton and its associated neutrino. For each of these decay modes, shown by the diagrams in Fig. 1, a dedicated event selection is performed to optimise the search significance, as detailed in Table 1.

The results of the searches are interpreted in simplified models [11–13] as a function of the top squark and lightest neutralino masses. Additionally, results are also interpreted in one phenomenological minimal supersymmetric standard model (pMSSM) [14–17] model including the following decay modes: $\tilde{t} \rightarrow t {\tilde{\chi }^0_1}$, $\tilde{t} \rightarrow b {\tilde{\chi }^\pm _1}$ with ${\tilde{\chi }^\pm _1} \rightarrow W {\tilde{\chi }^0_1}$ and $\tilde{t} \rightarrow t {\tilde{\chi }^0_2}$, with ${\tilde{\chi }^0_2} \rightarrow h/Z {\tilde{\chi }^0_1}$. Previous ATLAS [18, 19] and CMS [20–26–32] analyses have set exclusion limits at 95% confidence level (CL) on the signal scenarios considered here. When considering simplified models including the $\tilde{t} \rightarrow t {\tilde{\chi }^0_1}$ decay, top squark masses up to about 700 GeV have been excluded for a nearly massless lightest neutralino. For the same assumptions about the lightest neutralino mass, if the $\tilde{t} \rightarrow b {\tilde{\chi }^\pm _1}$ decay is dominant, top squark masses up to about 500 GeV have been excluded.

**Table 1:** Summary of the sections dedicated to the two-body, three-body and four-body selections and signal types targeted by each selection

	Two-body	Three-body	Four-body
Variables	Section 4.1		
Event selection	Section 4.2	Section 4.3	Section 4.4
Background determination	Section 6.1	Section 6.2	Section 6.3
Results	Section 8.1	Section 8.2	Section 8.3
Interpretation	Section 8.4		
Targeted decay modes	$b{\tilde{\chi }^\pm _1}$ and $t{\tilde{\chi }^0_1}$	$bW{\tilde{\chi }^0_1}$	$b \ell \nu {\tilde{\chi }^0_1}$
Signal diagram	Figure 1a, b	Figure 1c	Figure 1d
Targeted $m_{\tilde{t}}$ range	$> m_b+m_{{\tilde{\chi }^\pm _1}}$	$\ge m_b+m_W+ m_{{\tilde{\chi }^0_1}}$	$< m_b+m_W+ m_{{\tilde{\chi }^0_1}}$
	or $> m_t+m_{{\tilde{\chi }^0_1}}$	and $< m_t+m_{{\tilde{\chi }^0_1}}$	

## ATLAS detector

The ATLAS detector [33] at the LHC is a multi-purpose particle detector with a cylindrical forward–backward symmetric geometry^1^ and an approximate $4\pi$ coverage in solid angle. It consists of an inner tracking detector (ID) surrounded by a thin superconducting solenoid providing a 2 T axial magnetic field, electromagnetic and hadron calorimeters, and a muon spectrometer. The inner tracking detector covers the pseudorapidity range $|\eta | < 2.5$. It consists of silicon pixel, silicon microstrip, and transition radiation tracking detectors. The newly installed innermost layer of pixel sensors [34] was operational for the first time during the 2015 data-taking. Lead/liquid-argon (LAr) sampling calorimeters provide electromagnetic (EM) energy measurements with high granularity. A hadron (steel/scintillator-tile) calorimeter covers the central pseudorapidity range ($|\eta | < 1.7$). The end-cap and forward regions are instrumented with LAr calorimeters for both the EM and hadronic energy measurements up to $|\eta | = 4.9$. The muon spectrometer surrounds the calorimeters and features three large air-core toroid superconducting magnets with eight coils each. It includes a system of precision tracking chambers and fast detectors for triggering. The field integral of the toroids ranges between 2.0 and 6.0 Tm across most of the detector.

## Data samples and event reconstruction

The data were collected by the ATLAS detector in 2015 and 2016 during *pp* collisions at a centre-of-mass energy of $\sqrt{s} = 13$ TeV, with a peak instantaneous luminosity of $\mathcal{L} =1.4 \times 10^{34}~\hbox {cm}^{-2}\hbox {s}^{-1}$, a bunch spacing of 25 ns, and an average number of *pp* interactions per bunch crossing (pile-up) of $\langle \mu \rangle = 14$ in 2015 and $\langle \mu \rangle = 24$ in 2016. Only events taken in stable beam conditions, and for which all relevant detector systems were operational, are considered in this analysis. The integrated luminosity of the resulting data set is $36.1~\hbox {fb}^{-1}$, with an uncertainty of $\pm \,3.2$%. This uncertainty is derived, following a methodology similar to that detailed in Ref. [35], from a preliminary calibration of the luminosity scale using *x*–*y* beam-separation scans performed in August 2015 and May 2016.

Candidate events are required to have a reconstructed vertex with at least two associated tracks with transverse momentum $p_\mathrm{T} > 400~\hbox {MeV}$. The vertex with the highest scalar sum of the squared transverse momenta of the associated tracks is considered the primary vertex of the event.

Electron (*baseline*) candidates are reconstructed from three-dimensional electromagnetic calorimeter energy depositions matched to ID tracks, and are required to have pseudorapidity $|\eta |<2.47$, $p_\mathrm{T}>7\hbox { GeV}$, and to pass a loose likelihood-based identification requirement [36]. The likelihood input variables include measurements of calorimeter shower shapes and of track properties from the ID.

Muon (*baseline*) candidates are reconstructed in the pseudorapidity region $|\eta |<2.4$ from muon spectrometer tracks matching ID tracks. They must have $p_\mathrm{T}> 7\hbox { GeV}$ and must pass the medium identification requirements defined in Ref. [37], which are based on requirements on the number of hits in the different ID and muon spectrometer subsystems, and on the significance of the charge-to-momentum ratio (*q* / *p*) measurement  [37].

Jets are reconstructed from three-dimensional energy clusters in the calorimeter [38] with the anti-$k_t$ jet clustering algorithm [39, 40] with a radius parameter $R=0.4$. Only jet candidates with $p_\mathrm{T}>20\hbox { GeV}$ and $|\eta |<2.8$ are considered. Jets are calibrated as described in Refs. [41, 42], and the expected average energy contribution from pile-up clusters is subtracted according to the jet area [43]. Additional selections are applied to jets with $p_\mathrm{T}< 60 \hbox { GeV}$ and $|\eta |<2.4$ in order to reject jets produced in pile-up collisions [44]. The “medium” working point is used for the pile-up rejection, which has an efficiency of about 92% for jets produced by the hard scatter. Jets resulting from the hadronisation of *b*-quarks are identified using a multivariate *b*-tagging algorithm (MV2c10), which is based on quantities such as impact parameters of associated tracks and reconstructed secondary vertices [45, 46]. This algorithm is used at a working point that provides 77% *b*-tagging efficiency in simulated $t\bar{t}$ events, and a rejection factor of 134 for light-quark flavours and gluons and 6 for charm jets. The jets satisfying the *b*-tagging requirements are referred to as *b*-jets.

Events are discarded if they contain any jet with $p_\mathrm{T}>20 \hbox { GeV}$ failing to satisfy basic quality selection criteria that reject detector noise and non-collision backgrounds [47].

To resolve reconstruction ambiguities, an overlap removal algorithm is applied to candidate leptons and jets. Non-*b*-tagged jets which lie within $\Delta R =\sqrt{(\Delta y)^2+(\Delta \phi )^2} < 0.2$ (here *y* stands for the rapidity) from an electron candidate are removed, and the same is done for jets which lie close to a muon candidate and are consistent with the characteristics of jets produced by muon bremsstrahlung. Finally, any lepton candidate which lies within $\Delta R < 0.4$ from the direction of a surviving jet candidate is removed, in order to reject leptons from the decay of a *b*- or *c*-hadron. Electrons which share an ID track with a muon candidate are also removed.

Additional selections are then applied to the remaining lepton and jet candidates. Tighter requirements on the lepton candidates are imposed, which are then referred to as “signal” electrons or muons. Signal electrons must satisfy the *medium* likelihood-based identification requirement as defined in Ref. [36]. Signal electrons must have a transverse impact parameter with respect to the reconstructed primary vertex, $d_0$, with a significance of $\vert d_0\vert /\sigma (d_0) < 5$. For signal muons, the corresponding requirement is $\vert d_0\vert /\sigma (d_0) < 3$. The tracks associated with the signal leptons must have a longitudinal impact parameter with respect to the reconstructed primary vertex, $z_0$, satisfying $\vert z_0 \sin \theta \vert < 0.5$ mm. Isolation criteria are applied to both electrons and muons by placing an upper limit on the sum of the transverse energy of the calorimeter energy clusters in a cone of $\Delta R_\eta = \sqrt{(\Delta \eta )^2+(\Delta \phi )^2} = 0.2$ around the electron (excluding the deposit from the electron itself), and the scalar sum of the $p_\mathrm{T}$ of tracks within a variable-size cone around the lepton (excluding its own track). The track isolation cone radius for electrons (muons) is given by the smaller of $\Delta R = 10~\hbox {GeV}/p_\mathrm{T}$ and $\Delta R_\eta = 0.2\,(0.3)$. The isolation criteria are optimised such that the isolation selection efficiency is uniform across $\eta$, and it increases from 95% for $p_\mathrm{T} = 25~\hbox {GeV}$ to 99% for $p_\mathrm{T} = 60~\hbox {GeV}$ in $Z\rightarrow \ell \ell$ events.

Jets are required to have $| \eta | < 2.5$.

The missing transverse momentum ($\mathbf { p}^{\mathrm {miss}}_{\mathrm {T}}$), whose magnitude is denoted by $E_{\text {T}}^{\text {miss}}$, is defined as the negative vector sum of the transverse momenta of all identified baseline objects (electrons, muons, jets) and an additional soft term. The soft term is constructed from all tracks that are not associated with any reconstructed electron, muon or jet, but which are associated with the primary vertex. In this way, the $E_{\text {T}}^{\text {miss}}$ value is adjusted for the best calibration of the jets and the other identified objects above, while maintaining pile-up independence in the soft term [48, 49].

## Event selection

For the two-body and three-body selections, events are accepted if they pass an online selection (trigger) requiring a minimum of two electrons, two muons or an electron and a muon matched to the trigger objects. The offline selection requires that the leading lepton has a $p_\mathrm{T}$ larger than 25 GeV and the subleading lepton a $p_\mathrm{T}$ larger than 20 GeV, ensuring that trigger efficiencies are constant in the relevant phase space. The four-body selection accepts events passing an $E_{\text {T}}^{\text {miss}}$-based trigger and having offline $E_{\text {T}}^{\text {miss}}$ > 200 GeV. This ensures that the trigger efficiency is constant in the relevant phase space. Using this trigger permits the use of a reduced lepton $p_\mathrm{T}$ threshold of 7 GeV, increasing acceptance for the low lepton $p_\mathrm{T}$ produced in the four-body $\tilde{t} \rightarrow b\ell \nu {\tilde{\chi }^0_1}$ decay.

Events are required to have exactly two signal leptons which must be of opposite charge (electrons, muons, or one of each) with an invariant mass (regardless of the flavour of the leptons in the pair) $m_{\ell \ell }$ greater than $20~\hbox {GeV}$ ($10~\hbox {GeV}$ for the four-body selection) in order to remove leptons from low-mass resonances. Except for the four-body selection, events with same-flavour (SF) lepton pairs with $m_{\ell \ell }$ between 71.2 and 111.2 GeV are rejected, in order to reduce the backgrounds with leptons produced by *Z* bosons. No additional selection is applied to the $m_{\ell \ell }$ value of different-flavour (DF) lepton pairs. In the following, the requirements described in the preceding part of this section are referred to as “common selection”.

### Discriminators and kinematic variables

For the different decay modes considered, dedicated sets of discriminating variables are used to separate the signal from the SM backgrounds.

The missing transverse momentum and the $p_\mathrm{T}$ of the leading leptons and jets are used to define three useful ratio variables :

$$\begin{aligned} R_{2\ell 2j}= & {} E_{\mathrm{T}}^{\mathrm{miss}} / (E_{\mathrm{T}}^{\mathrm{miss}} + p_\mathrm{T}(\ell _1) + p_\mathrm{T}(\ell _2) + p_\mathrm{T}(j_1) + p_\mathrm{T}(j_2)),\\ R_{2\ell }= & {} E_{\mathrm{T}}^{\mathrm{miss}} / (p_\mathrm{T}(\ell _1) + p_\mathrm{T}(\ell _2)), \end{aligned}$$

and

$$\begin{aligned} R_{2\ell 4j} = {E_{\mathrm{T}}^{\mathrm{miss}}}/(E_{\mathrm{T}}^{\mathrm{miss}} + p_\mathrm{T}(\ell _1) + p_\mathrm{T}(\ell _2) + \sum _{i=1,\ldots ,N\le 4}p_\mathrm{T}(j_i)), \end{aligned}$$

where $p_\mathrm{T}(\ell _1)$ and $p_\mathrm{T}(\ell _2)$ are the leading and subleading lepton transverse momenta and $p_\mathrm{T}(j_{i =1,\ldots ,N\le 4})$ are the transverse momenta in decreasing order of up to the four leading jets. The variables $R_{2\ell 2j}$ and $R_{2\ell }$ are used to reject backgrounds, e.g. $Z/\gamma ^*+\mathrm {jets}$, which peak at lower values than the signal. Similarly, $R_{2\ell 4j}$ is a powerful discriminant against multi-jet events.

Other variables employed are :

- $\mathbf p^{\ell \ell }_{\mathrm {T,boost}}$: defined as the vector
  $$\begin{aligned} \mathbf p^{\ell \ell }_{\mathrm {T,boost}} = \mathbf { p}^{\mathrm {miss}}_{\mathrm {T}} + \mathbf {p}_\mathrm{T}(\ell _1) + \mathbf {p}_\mathrm{T}(\ell _2). \end{aligned}$$
  The $\mathbf p^{\ell \ell }_{\mathrm {T,boost}}$ variable, with magnitude $p^{\ell \ell }_{\mathrm {T,boost}}$, can be interpreted as the opposite of the vector sum of all the transverse hadronic activity in the event.
- $\Delta \phi _\mathrm {boost}$: the azimuthal angle between the $\mathbf { p}^{\mathrm {miss}}_{\mathrm {T}}$ vector and the $\mathbf p^{\ell \ell }_{\mathrm {T,boost}}$ vector.
- $$\Delta x$$: defined as
  $$\begin{aligned} \Delta x = \frac{2\cdot \left( p_{\text {z}} (\ell _1)+p_{\text {z}} (\ell _2)\right) }{E_{\text {C}M}} \end{aligned}$$
  where $E_{\text {C}M}=13$ TeV is used and $p_{\text {z}} (\ell _1)$,$p_{\text {z}} (\ell _2)$ are respectively the leading and subleading lepton longitudinal momenta. This variable helps to discriminate between gluon- and quark-initiated processes. The former tend to peak towards zero, while the latter tend to peak at higher values.
- $\cos \theta _b$: the cosine of the angle between the direction of motion of either of the two leptons and the beam axis in the centre-of-mass frame of the two leptons [50]. This variable is sensitive to the spin of the pair-produced particle, providing additional rejection against diboson backgrounds.
- $m_{\tiny {\text{ T2 }}}^{\ell \ell }$: lepton-based “stransverse” mass. The stransverse mass defined in Refs. [51, 52] is a kinematic variable used to bound the masses of a pair of identical particles which have each decayed into a visible and an invisible particle. This quantity is defined as
  $$\begin{aligned} m_{\tiny {\text{ T2 }}} ( \mathbf {p}_{\mathrm {T,1}}, \mathbf {p}_{\mathrm {T,2}}, \mathbf {q}_{\mathrm {T}})\\ = \min _{\mathbf {q}_{\mathrm {T,1}} + \mathbf {q}_{\mathrm {T,2}} = \mathbf {q}_{\mathrm {T}} } \left\{ \max [\; m_{\mathrm {T}}( \mathbf {p}_{\mathrm {T,1}}, \mathbf {q}_{\mathrm {T,1}} ), m_{\mathrm {T}}( \mathbf {p}_{\mathrm {T,2}}, \mathbf {q}_{\mathrm {T,2}} ) \;] \right\} , \end{aligned}$$
  where $m_{\mathrm T}$ indicates the transverse mass,^2^ $\mathbf {p}_{\mathrm {T,1}}$ and $\mathbf {p}_{\mathrm {T,2}}$ are the transverse momentum vectors of two particles, and $\mathbf {q}_{\mathrm {T,1}}$ and $\mathbf {q}_{\mathrm {T,2}}$ are transverse momentum vectors with $\mathbf {q}_{\mathrm {T}} = \mathbf {q}_{\mathrm {T,1}} + \mathbf {q}_{\mathrm {T,2}}$. The minimisation is performed over all the possible decompositions of $\mathbf {q}_{\mathrm {T}}$. For $t\bar{t}$ or *WW* decays with $t\rightarrow b \ell \nu$ and $W \rightarrow \ell \nu$, when the transverse momenta of the two leptons in each event are taken as $\mathbf {p}_{\mathrm {T,1}}$ and $\mathbf {p}_{\mathrm {T,2}}$, and $\mathbf { p}^{\mathrm {miss}}_{\mathrm {T}}$ as $\mathbf {q}_\mathrm {T}$, $m_T2 (\mathbf {p}_\mathrm{T}(\ell _1), \mathbf {p}_\mathrm{T}(\ell _2), \mathbf { p}^{\mathrm {miss}}_{\mathrm {T}})$ is bounded sharply from above by the mass of the *W* boson [53, 54]. In the $\tilde{t} \rightarrow b {\tilde{\chi }^\pm _1}$ decay mode the upper bound is strongly correlated with the mass difference between the chargino and the lightest neutralino. In this paper, $m_T2 (\mathbf {p}_\mathrm{T}(\ell _1), \mathbf {p}_\mathrm{T}(\ell _2), \mathbf { p}^{\mathrm {miss}}_{\mathrm {T}})$ is referred to simply as $m_{\tiny {\text{ T2 }}}^{\ell \ell }$.

The three-body selection uses a number of “super-razor” variables that are defined in Ref. [55]. They are designed to identify events with two massive parent particles (i.e. top squarks) each decaying into a set of visible (only leptons are considered in this case, all other particles including jets are ignored) and invisible particles (i.e. neutrinos and neutralinos). These variables are:

- $$R_{p_{\text {T}}}$$: defined as
  $$\begin{aligned} R_{p_{\text {T}}} = \frac{|\vec {J}_{\text {T}}|}{|\vec {J}_{\text {T}}| + \sqrt{\hat{s}}_{\text {R}}/4}, \end{aligned}$$
  where $\vec {J}_{\text {T}}$ is the vector sum of the transverse momenta of the visible particles and the missing transverse momentum, and $\sqrt{\hat{s}_{\text {R}}}$ is a measure of the system’s energy in the razor frame *R* as defined in Ref. [55] as the frame in which the two visible leptons have equal and opposite $p_{\text {z}}$. In the case where all possible visible particles are considered, the razor frame *R* becomes an approximation of the pair production centre-of-mass frame with the centre-of-mass energy $\sqrt{\hat{s}}_{\text {R}}$. In this analysis, only leptons are considered in the visible system. Therefore, $R_{p_{\text {T}}}$ tends towards zero in events that do not contain additional activity (i.e. dibosons) due to vanishing $|\vec {J}_{\text {T}}|$, whereas in events that contain additional activity (i.e. $t\bar{t}$) this variable tends towards unity, thus providing separation power between the two cases.
- $\gamma _\text {R+1}$: The Lorentz factor associated with the boosts from the razor frame *R* to the approximations of the two decay frames of the parent particles. It is a measure of how the two visible systems are distributed, tending towards unity when the visible particles are back-to-back or have different momenta, while preferring lower values when they are equal in momenta and collinear.
- $$M_{\Delta }^\text {R}$$: defined as
  $$\begin{aligned} M_{\Delta }^\text {R}\ = \frac{\sqrt{\hat{s}_{\text {R}}}}{\gamma _{{\text {R}}+1}}. \end{aligned}$$
  This variable has a kinematic end-point that is proportional to the mass-splitting between the parent particle and the invisible particle. Therefore, it provides rejection against both the top quark and diboson production processes when it is required to be greater than the mass of the *W* boson, and in this case it also helps to reject the residual $Z/\gamma ^*+\mathrm {jets}$ background.
- $\Delta \phi _{\beta }^\text {R}$: The quantity $\Delta \phi _{\beta }^\text {R}$ is the azimuthal angle between the razor boost from the laboratory to the *R* frame and the sum of the visible momenta as evaluated in the *R* frame. For systems where the invisible particle has a mass that is comparable to the pair-produced massive particle, this variable has a pronounced peak near $\pi$, making it, in general, a good discriminator in searches for models with small mass differences.

### Two-body event selection

This selection targets the top squark two-body decays (Fig. 1a, b) into either a bottom quark and a chargino, with the chargino decaying into the lightest neutralino and a *W* boson, or a near-mass-shell top quark and a neutralino.

In these decays, the kinematic properties of signal events are similar to those of $t\bar{t}$ events. In particular, when the top squarks are produced at rest the momenta carried by the neutralinos in the final state are small and the discrimination difficult. Better separation between signal events and the $t\bar{t}$ background can be obtained for top squark pairs which recoil from initial-state radiation (ISR).

Three signal regions (SRs), summarised in Table 2 and denoted by $\mathrm {SR(A,B,C)}^{\mathrm {2-body}}_{x}$, where *x* stands for the lower bound of the $m_{\tiny {\text{ T2 }}}^{\ell \ell }$ interval, were optimised to target different scenarios:

- $\text {SRA}^{\text {2-body}}_{180}$ targets the decays into $b {\tilde{\chi }^\pm _1}$ in scenarios where $m_{\tilde{t}_1}-m_{{\tilde{\chi }^\pm _1}}$ is below 10 GeV and the *b*-jets from the decay of the $\tilde{t}_1$ are too low in energy to be reconstructed. For this reason, *b*-jets with $p_\mathrm{T}>25$ GeV are vetoed to reduce the contamination from SM processes including top quarks. No further requirement is imposed on the hadronic activity of the event. Events with SF leptons are required to have $m_{\ell \ell } >111.2$ GeV and $R_{2\ell 2j} >0.3$ to reduce the contamination from $Z/\gamma ^*+\mathrm {jets}$ events. The contribution from diboson production is expected to be the dominant background in the SR and it is reduced by requiring the events to have $\Delta x <0.07$. Furthermore, events are required to have $m_{\tiny {\text{ T2 }}}^{\ell \ell } >180$ GeV.
- $\text {SRB}^{\text {2-body}}_{140}$ targets the decays into $b {\tilde{\chi }^\pm _1}$ in scenarios with a mass-splitting between the top squark and the chargino larger than 10 GeV, such that the jets from the hadronisation of *b*-quarks are expected to be detectable. At least two jets with $p_\mathrm{T}>25$ GeV are required, with at least one of them being identified as a *b*-jet. Events from $t\bar{t}$ and $Z/\gamma ^*+\mathrm {jets}$ production are suppressed by requiring $\Delta \phi _\mathrm {boost} <1.5$. The main expected SM processes satisfying this selection are $t\bar{t}$ and $t\bar{t}+Z$ with the $Z$ boson decaying into neutrinos. A final selection of $m_{\tiny {\text{ T2 }}}^{\ell \ell } >140$ GeV is applied. Because of the similar final state, this selection is the most sensitive to signal scenarios in which the $\tilde{t}_1$ decays into $t+{\tilde{\chi }^0_1}$, with large $m_{\tilde{t}_1} - m_{{\tilde{\chi }^0_1}}$.
- $\text {SRC}^{\text {2-body}}_{110}$ targets the decays into $t+{\tilde{\chi }^0_1}$, in scenarios where $m_{\tilde{t}_1} \sim m_{{\tilde{\chi }^0_1}}+m_{t}$. Candidate events are required to have $E_{\text {T}}^{\text {miss}} > 200$ GeV and at least three jets with $p_\mathrm{T}>25$ GeV, where one of the jets is interpreted as ISR. The other two jets are expected to arise from the decay of the top quarks in the final state. One of the jets in the event is required to be *b*-tagged, effectively separating the signal events from SM diboson production. The $Z/\gamma ^*+\mathrm {jets}$ background is suppressed by requiring $R_{2\ell }$ to be larger than 1.2. Events are finally required to have $m_{\tiny {\text{ T2 }}}^{\ell \ell } >110$ GeV.

For the model-dependent exclusion limits, a shape fit of the $m_{\tiny {\text{ T2 }}}^{\ell \ell }$ distribution is performed for the $\text {SRA}^{\text {2-body}}_{180}$ and $\text {SRB}^{\text {2-body}}_{140}$ selections: the distribution is divided into bins of width 20 GeV, starting from $m_{\tiny {\text{ T2 }}}^{\ell \ell } =120$ GeV; the last bin’s low boundary corresponds to the requirement on the same variable in the definitions of $\text {SRA}^{\text {2-body}}_{180}$ and $\text {SRB}^{\text {2-body}}_{140}$; each bin is referred to as $\mathrm {SR(A,B)}^{\mathrm {2-body}}_{x,y}$, where *x* and *y* denote the low and high edges of the bin.

**Table 2:** *Two-body selection* signal region definitions

	$\text {SRA}^{\text {2-body}}_{180}$		$\text {SRB}^{\text {2-body}}_{140}$		$\text {SRC}^{\text {2-body}}_{110}$	
Lepton flavour	SF	DF	SF	DF	SF	DF
$p_\mathrm{T}(\ell _1),p_\mathrm{T}(\ell _2)$ [GeV]	$> 25$, $> 20$		$> 25$, $> 20$		$> 25$, $> 20$	
			[20, 71.2]		[20, 71.2]	
$m_{\ell \ell }$ [GeV]	$> 111.2$	$> 20$	or	$> 20$	or	$> 20$
			$> 111.2$		$> 111.2$	
$R_{2\ell 2j}$	$> 0.3$	$-$	$-$		$-$	
$R_{2\ell }$	$-$		$-$		$>1.2$	
$\Delta x$	$< 0.07$		$-$		$-$	
$\Delta \phi _\mathrm {boost}$	$-$		$< 1.5$		$-$	
$n_{\mathrm {jets}}$	$-$		$\ge 2$		$\ge 3$	
$n_{b\text {-jets}}$	$= 0$		$\ge 1$		$\ge 1$	
$E_{\text {T}}^{\text {miss}}$ [GeV]	$-$		$-$		$> 200$	
$m_{\tiny {\text{ T2 }}}^{\ell \ell }$ [GeV]	$> 180$		$> 140$		$> 110$	

### Three-body event selection

This selection targets the top squark three-body decay mode (Fig. 1c), which is expected to be the dominant decay mode when the two-body decay mode into the lightest chargino or neutralino is kinematically forbidden, i.e. for $m_{{\tilde{\chi }^0_1}}+m_{W}+m_{b}< m_{\tilde{t}_1} < m_{{\tilde{\chi }^0_1}} + m_{t}$ and $m_{\tilde{t}_1} < m_{{\tilde{\chi }^\pm _1}} + m_{b}$.

Two orthogonal signal regions, SR$^{\text {3-body}}_{W}$ and SR$^{\text {3-body}}_{t}$, are summarised in Table 3. The SR$^{\text {3-body}}_{W}$ targets the region where $\Delta m(\tilde{t},{\tilde{\chi }^0_1}) \sim m_{W}$ in which the produced *b*-jets have low transverse momentum, and hence are often not reconstructed. The second signal region SR$^{\text {3-body}}_{t}$ targets the region in which $\Delta m(\tilde{t},{\tilde{\chi }^0_1}) \sim m_{t}$.

The two regions make use of a common set of requirements on $R_{p_{\text {T}}}$, $\gamma _\text {R+1}$, and in the two-dimensional ($\cos \theta _b$, $\Delta \phi _{\beta }^\text {R}$) plane. In addition, SR$^{\text {3-body}}_{W}$ requires that no *b*-jet is identified in the event and that $M_{\Delta }^\text {R}$  $>95$ GeV. The large $M_{\Delta }^\text {R}$ requirement suppresses the top quark and diboson backgrounds. In the case of SR$^{\text {3-body}}_{t}$, the requirements are: at least one *b*-jet and $M_{\Delta }^\text {R}$ $>110$ GeV. The *b*-jet requirement makes the selection orthogonal to SR$^{\text {3-body}}_{W}$, so that the two SRs can be statistically combined. Furthermore, a slightly tighter $M_{\Delta }^\text {R}$ requirement is necessary to eliminate the background that originates from top quark production processes.

**Table 3:** *Three-body selection* signal region definitions

	SR$^{\text {3-body}}_{W}$		SR$^{\text {3-body}}_{t}$	
Lepton flavour	SF	DF	SF	DF
$p_\mathrm{T}(\ell _1),p_\mathrm{T}(\ell _2)$ [GeV]	$>25$, $>20$		$>25$, $>20$	
	[20, 71.2]		[20, 71.2]	
$m_{\ell \ell }$ [GeV]	or	$> 20$	or	$> 20$
	$> 111.2$		$> 111.2$	
$n_{b\text {-jets}}$	$=0$		$\ge 1$	
$M_{\Delta }^\text {R}$ [GeV]	$>95$		$>110$	
$R_{p_{\text {T}}}$	$>0.7$		$>0.7$	
1/$\gamma _\text {R+1}$	$>0.7$		$>0.7$	
$\Delta \phi _{\beta }^\text {R}$	$> 0.9 |\cos \theta _b | + 1.6$		$> 0.9 |\cos \theta _b | + 1.6$	

### Four-body event selection

The selection described here targets the four-body decay mode of the top squark (Fig. 1d) for scenarios where $m_{\tilde{t}_1} < m_{{\tilde{\chi }^0_1}} + m_{b} + m_{W}$ and $m_{\tilde{t}_1} < m_{{\tilde{\chi }^\pm _1}} + m_{b}$. In this region the top squark decay into $c{\tilde{\chi }^0_1}$ might be dominant, depending on various SUSY model parameters. The branching ratio into this final state is here assumed to be negligible. For these small mass splittings, the leptons in the final state, originating from the virtual *W* boson decays, are expected to have low $p_\mathrm{T}$.

Signal events can be distinguished from SM processes if a high-$p_\mathrm{T}$ jet from ISR leads to a large transverse boost of the sparticle pair system and enhances the $E_{\text {T}}^{\text {miss}}$ value. At least two jets with $p_\mathrm{T}>25~\hbox {GeV}$ are required in the event. The leading jet is considered to be the ISR jet and required to have $p_\mathrm{T}>150$ GeV. Since the jets resulting from $\tilde{t}$ decays tend to have low $p_\mathrm{T}$ in this scenario, at most one more energetic jet with $p_\mathrm{T}>25~GeV$ is permitted in the event and the transverse momentum of the third jet (if present) must satisfy $p_\mathrm{T}(j_3)/E_{\text {T}}^{\text {miss}} < 0.14$.

In order to remove events originating from low-mass resonances, the invariant mass of the two leptons, $m_{\ell \ell }$, is required to be greater than 10 GeV. Furthermore, upper limits on $p_{\text {T}} (\ell _1)$ and $p_{\text {T}} (\ell _2)$, respectively of 80 GeV and 35 GeV, are applied.

The signal region $\text {SR}^{\mathrm {4-body}}$ is defined as summarised in Table 4. The two variables $R_{2\ell 4j}$ and $R_{2\ell }$ must be larger than 0.35 and 12 to reject multi-jet and $t\bar{t}$ backgrounds, respectively. Finally, the two most energetic jets in the event must not be tagged as *b*-jets.

**Table 4:** *Four-body selection* signal region definition

	$$\text {SR}^{\mathrm {4-body}}$$
Lepton flavour	SF and DF
$E_{\text {T}}^{\text {miss}}$ [GeV]	$> 200$
$$p_\mathrm{T}(\ell _1)$$ [GeV]	[7, 80]
$$p_\mathrm{T}(\ell _2)$$ [GeV]	[7, 35]
$m_{\ell \ell }$ [GeV]	$> 10$
$n_{\mathrm {jets}}$	$\ge 2$
$p_\mathrm{T}(j_1)$ [GeV]	$> 150$
$p_\mathrm{T}(j_2)$ [GeV]	$> 25$
$p_\mathrm{T}(j_3)/E_{\text {T}}^{\text {miss}}$	$< 0.14$
$R_{2\ell 4j}$	$> 0.35$
$R_{2\ell }$	$> 12$
$n_{b\text {-jets}}$	veto on $j_1$ and $j_2$

## Samples of simulated events

Monte Carlo (MC) simulated event samples are used to aid in the estimation of the background from SM processes and to model the SUSY signal. The event generator, parton shower and hadronisation generator, cross-section normalisation, parton distribution function (PDF) set and underlying-event parameter set (tune) of these samples are given in Table 5, and more details of the event generator configurations can be found in Refs. [56–59]. Cross-sections calculated at next-to-next-to-leading order (NNLO) in QCD including resummation of next-to-next-to-leading logarithmic (NNLL) soft gluon terms were used for top quark production processes. For production of top quark pairs in association with vector or Higgs bosons, cross-sections calculated at next-to-leading order (NLO) were used, and the event generator cross-sections calculated by Sherpa (at NLO for most of the processes) are used when normalising the multi-boson backgrounds. In all MC samples, except those produced by Sherpa, the EvtGen v1.2.0 program [60] was used to model the properties of the bottom and charm hadron decays. Additional MC samples are used when estimating systematic uncertainties, as detailed in Sect. 7.

**Table 5:** Simulated signal and background event samples: the corresponding event generator, parton shower generator, cross-section normalisation, PDF set and underlying-event tune are shown

Physics process	Event generator	Parton shower generator	Cross-section normalisation	PDF set	Tune
SUSY Signals	MadGraph5_aMC@NLO 2.2.3 [61]	Pythia 8.186 [62]	NLO + NLL [63–68]	NNPDF23LO [69]	A14 [70]
$Z/\gamma ^*+\mathrm {jets}$	Sherpa 2.2.1 [71]	Sherpa 2.2.1	NNLO [72]	NLO CT10 [69]	Sherpa default
$t\bar{t}$	powheg-box v2 [73]	Pythia 6.428 [74]	NNLO + NNLL [75–80]	NLO CT10	Perugia2012 [81]
*Wt*	powheg-box v2	Pythia 6.428	NNLO + NNLL [82]	NLO CT10	Perugia2012
$t\bar{t} W/Z/\gamma ^{*}$	MadGraph5_aMC@NLO 2.2.2	Pythia 8.186	NLO [61]	NNPDF23LO	A14
Diboson	Sherpa 2.2.1	Sherpa 2.2.1	Generator NLO	NLO CT10	Sherpa default
$t\bar{t}h$	MadGraph5_aMC@NLO 2.2.2	Herwig 2.7.1 [83]	NLO [84]	CTEQ6L1 [85]	A14
*Wh*, *Zh*	MadGraph5_aMC@NLO 2.2.2	Pythia 8.186	NLO [84]	NNPDF23LO	A14
$t\bar{t} WW$, $t\bar{t}t\bar{t}$	MadGraph5_aMC@NLO 2.2.2	Pythia 8.186	NLO [61]	NNPDF23LO	A14
*tZ*, *tWZ*, $t\bar{t}t$	MadGraph5_aMC@NLO 2.2.2	Pythia 8.186	LO	NNPDF23LO	A14
Triboson	Sherpa 2.2.1	Sherpa 2.2.1	Generator LO, NLO	CT10	Sherpa default

SUSY signal samples were generated from leading-order (LO) matrix elements with up to two extra partons, using the MadGraph5_aMC@NLO  [61] event generator. The two-body signals used Pythia 8.186 [62] for the modelling of the SUSY decay chain, parton showering, hadronisation and the description of the underlying event. The three-body and four-body signals were decayed with Pythia8 + MadSpin [86] instead. Parton luminosities were provided by the NNPDF23LO PDF set. Jet–parton matching was realised following the CKKW-L prescription [87], with a matching scale set to one quarter of the pair-produced superpartner mass. In all cases, the mass of the top quark was fixed at 172.5 GeV. Signal cross-sections were calculated to next-to-leading order in the strong coupling constant, adding the resummation of soft gluon emission at next-to-leading-logarithmic accuracy (NLO + NLL) [67, 88, 89]. The nominal cross-sections and their uncertainties were taken from an envelope of cross-section predictions using different PDF sets and factorisation and renormalisation scales, as described in Ref. [68]. All two-, three- and four-body samples were generated assuming a 100% branching ratio into the respective final states.

For the pMSSM inspired models, the mass spectrum of sparticles was calculated using Softsusy 3.7.3 [90] and cross-checked with SPheno 3.3.8 [91, 92] and Suspect 2.5 [93]. Hdecay and Sdecay, included in Susy-Hit [94] were used to generate decay tables of the SUSY particles.

To simulate the effects of additional *pp* collisions in the same and nearby bunch crossings, additional interactions were generated using the soft QCD processes of Pythia 8.186 with the A2 tune [95] and the MSTW2008LO PDF set [96], and they were overlaid onto each simulated hard-scatter event. The MC samples were reweighted to the pile-up distribution observed in the data. The MC samples were processed through an ATLAS detector simulation [97] based on Geant4 [98] or, in the case of $t\bar{t} t$ and the SUSY signal samples, a fast simulation using a parameterisation of the calorimeter response and Geant4 for the other parts of the detector [99]. All MC samples are reconstructed in the same manner as the data. Corrections derived from data control samples are applied to simulated events to account for differences between data and simulation in reconstruction efficiencies, momentum scale and resolution of leptons and in the efficiency and false positive rate for identifying jets resulting from the hadronisation of *b*-quarks.

## Background estimation

The dominant SM background processes satisfying the SR requirements are estimated by simulation, which is normalised to data and verified in separate regions of the phase space. Dedicated control regions (CRs), described in Sects. 6.1–6.3, enhanced in a particular background component are used for the normalisation. Subdominant background yields are taken directly from MC simulation or from additional independent studies in data. For each signal region, a simultaneous “background fit” is performed to the number of events found in the CRs, using a statistical minimisation based on a likelihood implemented in the HistFitter package [100]. In each fit, the normalisations of the background contributions having dedicated CRs are allowed to float, while the MC simulation is used to describe the shape of distributions of kinematical variables. The level of agreement between the background prediction and data is compared in dedicated validation regions (VRs), which are not used to constrain the background normalisation or nuisance parameters in the fit.

In order to keep the background control region kinematically as close as possible to the SR, the two-body, three-body and four-body selections use different sets of CRs. The definitions of the regions used in each analysis and the results of the fits are described in the following subsections.

The background due to jets misidentified as leptons (hereafter referred to as “fake” leptons) and non-prompt leptons is collectively referred to as “FNP”: it consists of semileptonic $t\bar{t}$, *s*-channel and *t*-channel single-top-quark, *W* + jets and light- and heavy-flavour multi-jet events. It is estimated from data with a method similar to that described in Refs. [101, 102]. Two types of lepton identification criteria are defined for this evaluation: “tight” and “loose”, corresponding to signal and baseline leptons described in Sect. 3. The method makes use of the number of observed events containing loose–loose, loose–tight, tight–loose and tight–tight lepton pairs in a given SR. The probability for prompt leptons satisfying the loose selection criteria to also pass the tight selection is measured using a $Z\rightarrow \ell \ell$ ($\ell = e, \mu$) sample. The equivalent probability for fake or non-prompt leptons is measured in data from multi-jet- and $t\bar{t}$-enriched control samples. The number of events containing a contribution from one or two fake or non-prompt leptons is calculated from these probabilities.

Systematic uncertainties in the samples of simulated events affect the expected yields in the different regions and are taken into account to determine the uncertainties in the background predictions. The systematic uncertainties are described by nuisance parameters, which are not constrained by the fit, since the number of floating background normalisation parameters is equal to the number of CRs. Each uncertainty source is described by a single nuisance parameter, and all correlations between background processes and selections are taken into account. A list of systematic uncertainties considered in the fits is provided in Sect. 7.

### Two-body selection background determination

The main background sources for the two-body selection are respectively diboson production in $\text {SRA}^{\text {2-body}}_{180}$and $t\bar{t}$ and $t\bar{t}$ + Z in $\text {SRB}^{\text {2-body}}_{140}$ and $\text {SRC}^{\text {2-body}}_{110}$. These processes are normalised to data in dedicated CRs, summarised in Table 6 together with the corresponding VRs: $\text {CR}^{\text {2-body}}_{t\bar{t}}$ (included in the background fits of $\text {SRA}^{\text {2-body}}_{180}$ and $\text {SRB}^{\text {2-body}}_{140}$), $\text {CR}^{\text {2-body}}_{t\bar{t},3j}$ (included in the background fit of $\text {SRC}^{\text {2-body}}_{110}$), $\text {CR}^{\text {2-body}}_{VV\text {-SF}}$ (included in the background fits of $\text {SRA}^{\text {2-body}}_{180}$ and $\text {SRB}^{\text {2-body}}_{140}$), $\text {CR}^{\text {2-body}}_{t\bar{t} Z}$ (included in the background fits of $\text {SRA}^{\text {2-body}}_{180}$, $\text {SRB}^{\text {2-body}}_{140}$ and $\text {SRC}^{\text {2-body}}_{110}$) and $\text {CR}^{\text {2-body}}_{VZ}$ (included in the background fits of $\text {SRA}^{\text {2-body}}_{180}$ and $\text {SRB}^{\text {2-body}}_{140}$). The control and validation regions are labelled using the targeted background process as subscript, which can also include additional selection details, and the associated selection as superscript. For example, the “3*j*” subscript of $\text {CR}^{\text {2-body}}_{t\bar{t},3j}$refers to the minimum jet multiplicity which is required in this control region. In $\text {CR}^{\text {2-body}}_{t\bar{t} Z}$ and $\text {CR}^{\text {2-body}}_{VZ}$, events with three charged leptons including one same-flavour opposite-charge pair with $|m_{\ell \ell }-m_{Z}| < 20$ GeV are selected. In order to mimic the kinematics of the $t\bar{t}$ + *Z* events with invisible *Z* decays, a corrected $E_{\text {T}}^{\text {miss}}$ variable, $E_{\mathrm {T,corr}}^{\mathrm {miss}}$, is defined by vectorially adding the momentum of the same-flavour opposite-charge lepton pair to the $\mathbf { p}^{\mathrm {miss}}_{\mathrm {T}}$ vector.

In order to test the reliability of the background prediction, the results of the simultaneous fit are cross-checked in VRs which are disjoint from both the corresponding control and signal regions. Overlapping regions, e.g. $\text {CR}^{\text {2-body}}_{t\bar{t}}$ and $\text {CR}^{\text {2-body}}_{t\bar{t},3j}$, are only included in independent background fits, so that no correlation is introduced. The expected signal contamination in the CRs is generally below 5%. The highest signal contamination in the VRs, of about $18\%$, is expected in $\text {VR}^{\text {2-body}}_{t\bar{t},3j}$ for a top squark mass of 400 GeV and a lightest neutralino mass of 175 GeV.

**Table 6:** *Two-body selection* control and validation regions definition. The common selection defined in Sect. 4 also applies to all regions except $\text {CR}^{\text {2-body}}_{t\bar{t} Z}$ and $\text {CR}^{\text {2-body}}_{VZ}$, which require three leptons including one same-flavour opposite-charge pair with $|m_{\ell \ell }-m_{Z}| < 20$ GeV

	$\text {CR}^{\text {2-body}}_{t\bar{t}}$	$\text {CR}^{\text {2-body}}_{t\bar{t},3j}$	$\text {CR}^{\text {2-body}}_{VV\text {-SF}}$	$\text {CR}^{\text {2-body}}_{t\bar{t} Z}$	$\text {CR}^{\text {2-body}}_{VZ}$	$\text {VR}^{\text {2-body}}_{t\bar{t}}$	$\text {VR}^{\text {2-body}}_{t\bar{t},3j}$	$\text {VR}^{\text {2-body}}_{VV\text {-DF}}$
Leptons	2, DF	2	2, SF	3	3	2, DF	2	2, DF
$m_{\tiny {\text{ T2 }}}^{\ell \ell }$ [GeV]	[100, 120]	[60, 100]	[100, 120]	$-$	$-$	$> 120$	$> 100$	[100, 120]
$$\begin{array}{l}n_{b\text {-jets}} \\ n_{\mathrm {jets}} \end{array}$$	$$\begin{array}{c} \ge 1 \\ \mathrm {-} \end{array}$$	$$\begin{array}{c} \ge 1 \\ \ge 3 \end{array}$$	$$\begin{array}{c} 0 \\ \mathrm {-} \end{array}$$	$$\begin{array}{c} \ge 2 \\ \ge 3 \end{array}$$ or $$\begin{array}{c} = 1 \\ \ge 4 \end{array}$$	$$\begin{array}{c} 0 \\ \mathrm {-} \end{array}$$	$$\begin{array}{c} \ge 1 \\ \ge 2 \end{array}$$	$$\begin{array}{c} \ge 1 \\ \ge 3 \end{array}$$	$$\begin{array}{c} 0 \\ \mathrm {-} \end{array}$$
$p^{\ell \ell }_{\mathrm {T,boost}}$ [GeV]	$-$	$-$	$< 25$	$-$	$-$	$-$	$-$	$< 25$
$\Delta \phi _\mathrm {boost}$	$-$	$-$	$-$	$-$	$-$	$> 1.5$	$-$	$-$
$R_{2\ell 2j}$	$-$	$-$	$> 0.3$	$-$	$-$	$-$	$-$	$-$
$E_{\mathrm {T,corr}}^{\mathrm {miss}}$ [GeV]	$-$	$-$	$-$	$> 120$	$> 120$	$-$	$-$	$-$
$E_{\text {T}}^{\text {miss}}$ [GeV]		$> 200$	$-$	$-$	$-$	$-$	$>200$	$-$
$R_{2\ell }$	$-$	$<1.2$	$-$	$-$	$-$	$-$	$<1.2$	$-$

**Fig. 2:**
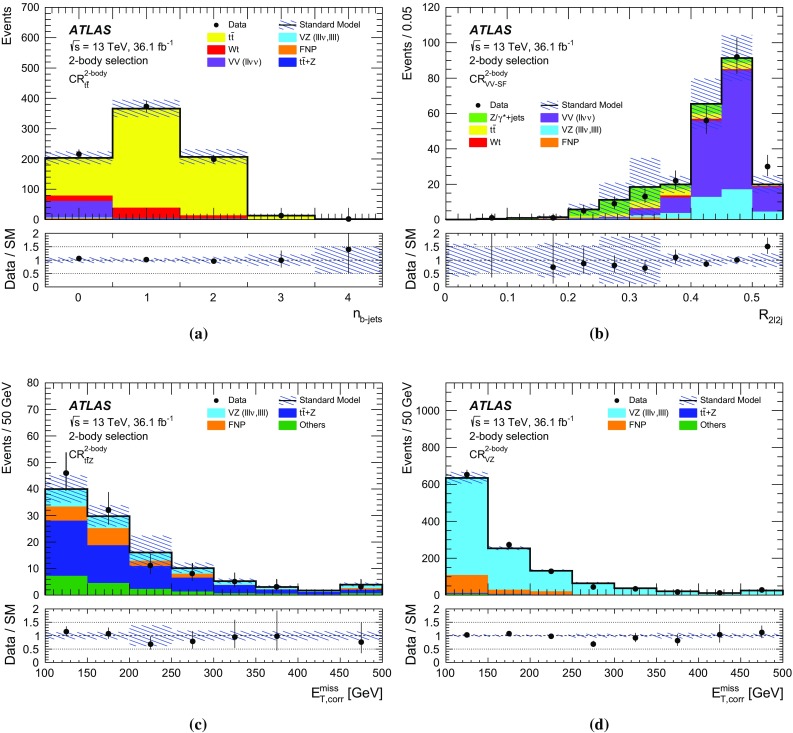
*Two-body selection* distributions of **a** $n_{b\text {-jets}}$ in $\text {CR}^{\text {2-body}}_{t\bar{t}}$, **b** $R_{2\ell 2j}$ in $\text {CR}^{\text {2-body}}_{VV\text {-SF}}$ and **c**, **d** $E_{\mathrm {T,corr}}^{\mathrm {miss}}$ in $\text {CR}^{\text {2-body}}_{t\bar{t} Z}$ and $\text {CR}^{\text {2-body}}_{VZ}$ after the $\text {SRA}^{\text {2-body}}_{180}$ background fit. The contributions from all SM backgrounds are shown as a histogram stack; the hatched bands represent the total uncertainty in the background predictions after the fit to the data has been performed. The counting uncertainty on data is also shown by the black error bars. The rightmost bin of each plot includes overflow events

Figure 2 shows the distributions of some of the kinematic variables used to define the four control regions after the $\text {SRA}^{\text {2-body}}_{180}$ background fit, so that the plots illustrate the modelling of the shape of each variable. In general, good agreement is found between the data and the background model within uncertainties. The other selection variables are equally well described by the background prediction.

The results of the background fits, as well as the MC expected background composition before the fit, are reported in Table 7 for the CRs used in the $\text {SRA}^{\text {2-body}}_{180}$ and $\text {SRB}^{\text {2-body}}_{140}$ background fits, and in Table 8 for the CRs used in the $\text {SRC}^{\text {2-body}}_{110}$ background fit. The normalisations for fitted backgrounds are found to be consistent with the theoretical predictions, when uncertainties are considered. By construction, in the CRs the yields observed and predicted by the fits are the same. Good agreement, within one standard deviation from the SM background prediction, is observed in the VRs and summarised in Fig. 5.

**Table 7:** *Two-body selection* background fit results for the CRs of the $\text {SRA}^{\text {2-body}}_{180}$ and $\text {SRB}^{\text {2-body}}_{140}$ background fits. The nominal predictions from MC simulation, are given for comparison for those backgrounds ($t\bar{t}$, *VV*-SF, $t\bar{t} Z$ and *VZ*) that are normalised to data in dedicated CRs. The “Others” category contains the contributions from $t\bar{t} W$, $t\bar{t} h$, $t\bar{t} WW$, $t\bar{t} t$, $t\bar{t} t\bar{t}$, *Wh*, *ggh* and *Zh* production. Combined statistical and systematic uncertainties are given. Entries marked “–” indicate a negligible background contribution

	$\text {CR}^{\text {2-body}}_{t\bar{t}}$	$\text {CR}^{\text {2-body}}_{VV\text {-SF}}$	$\text {CR}^{\text {2-body}}_{t\bar{t} Z}$	$\text {CR}^{\text {2-body}}_{VZ}$
Observed events	587	213	91	836
Estimated SM events	$587 \pm 24$	$213 \pm 15$	$91 \pm 10$	$836 \pm 29$
$t\bar{t}$	$532 \pm 25$	$14 \pm 4$	$-$	$-$
*Wt*	$44 \pm 6$	$4.0 \pm 1.5$	$-$	$-$
$Z/\gamma ^*+\mathrm {jets}$	$0.02_{-0.02}^{+0.05}$	$19 \pm 10$	$-$	$-$
*VV*-SF	$-$	$135 \pm 18$	$-$	$-$
*VV*-DF	$2.2 \pm 0.8$	$-$	$-$	$-$
*VZ*	$0.18 \pm 0.12$	$38 \pm 7$	$17.5 \pm 2.5$	$730 \pm 50$
$t\bar{t}+Z$	$2.2 \pm 0.8$	$0.07 \pm 0.07$	$47 \pm 12$	$8.9 \pm 2.5$
Others	$3.8 \pm 0.4$	$0.41 \pm 0.18$	$14.5 \pm 1.4$	$10.3 \pm 0.9$
Fake and non-prompt	$1.6 \pm 0.9$	$0_{-0}^{+5}$	$12 \pm 7$	$86 \pm 34$
Nominal MC, $t\bar{t}$	504	14	$-$	$-$
Nominal MC, *VV*-SF	$-$	122	$-$	$-$
Nominal MC, *VZ*	0.18	39	18	735
Nominal MC, $t\bar{t} + Z$	3.57	0.08	56	11

**Table 8:** *Two-body selection* background fit results for the CRs of the $\text {SRC}^{\text {2-body}}_{110}$ background fit. The nominal predictions from MC simulation, are given for comparison for those backgrounds ($t\bar{t}$ and $t\bar{t} Z$) that are normalised to data in dedicated CRs. The “Others” category contains the contributions from $t\bar{t} W$, $t\bar{t} h$, $t\bar{t} WW$, $t\bar{t} t$, $t\bar{t} t\bar{t}$, *Wh*, *ggh* and *Zh* production. Combined statistical and systematic uncertainties are given. Entries marked “–” indicate a negligible background contribution

	$\text {CR}^{\text {2-body}}_{t\bar{t},3j}$	$\text {CR}^{\text {2-body}}_{t\bar{t} Z}$
Observed events	212	91
Estimated SM events	$212 \pm 15$	$91 \pm 10$
$t\bar{t}$	$184 \pm 16$	$-$
$t\bar{t}$ + Z	$1.03 \pm 0.32$	$47 \pm 12$
*Wt*	$23 \pm 7$	$-$
*VV*	$1.69 \pm 0.30$	$17.7 \pm 2.2$
$Z/\gamma ^*+\mathrm {jets}$	$0.05 \pm 0.02$	$-$
Others	$1.91 \pm 0.12$	$14.6 \pm 1.0$
Fake and non-prompt	$-$	$12 \pm 7$
Nominal MC, $t\bar{t}$	201	$-$
Nominal MC, $t\bar{t}+\hbox {Z}$	1.23	55.7

### Three-body selection background determination

In the three-body signal regions defined in Sect. 4.3, the SM background is dominated by diboson and $t\bar{t}$ production. A single control region is used for $t\bar{t}$ production, while two CRs are defined to target diboson events with either same-flavour or different-flavour lepton pairs. The background predictions are tested in VRs that are defined to be kinematically adjacent to, yet disjoint from, the signal regions. The definitions of the control and validation regions are shown in Table 9. The overlap between $\text {VR}^{\text {3-body}}_{t\bar{t}}$ and $\text {VR}^{\text {3-body}}_{VV\text {-DF}}$ does not affect the final results as these regions are not used to constrain the background normalisations. The signal contamination in the CRs and VRs is generally small, with the maximum found to be about 12% in $\text {VR}^{\text {3-body}}_{VV\text {-DF}}$ for a top squark mass of 220 GeV and a lightest neutralino mass of 110 GeV.

**Table 9:** *Three-body selection* control and validation regions definitions. The common selection defined in Sect. 4 also applies to all regions

	$\text {CR}^{\text {3-body}}_{t\bar{t}}$	$\text {CR}^{\text {3-body}}_{VV\text {-DF}}$	$\text {CR}^{\text {3-body}}_{VV\text {-SF}}$	$\text {VR}^{\text {3-body}}_{t\bar{t}}$	$\text {VR}^{\text {3-body}}_{VV\text {-DF}}$	$\text {VR}^{\text {3-body}}_{VV\text {-SF}}$
Lepton flavour	DF	DF	SF	DF	DF	SF
$|m_{\ell \ell }-m_{Z}|$ [GeV]	$-$	$-$	$> 20$	$-$	$-$	$>20$
$n_{b\text {-jets}}$	$>0$	$=0$	$=0$	$=0$	$=0$	$=0$
$M_{\Delta }^\text {R}$ [GeV]	$>80$	$>50$	$>70$	$>80$	[50, 95]	[60, 95]
$R_{p_{\text {T}}}$	$>0.7$	$<0.5$	$<0.5$	$<0.7$	$<0.7$	$<0.4$
1/$\gamma _\text {R+1}$	$-$	$>0.7$	$>0.7$	$-$	$>0.7$	$>0.7$
($\cos \theta _b$, $\Delta \phi _{\beta }^\text {R}$)	$\Delta \phi _{\beta }^\text {R} <(0.9\times |\cos \theta _b |+1.6)$			$\,\,\Delta \phi _{\beta }^\text {R} >(0.9\times |\cos \theta _b |+1.6)$		

**Fig. 3:**
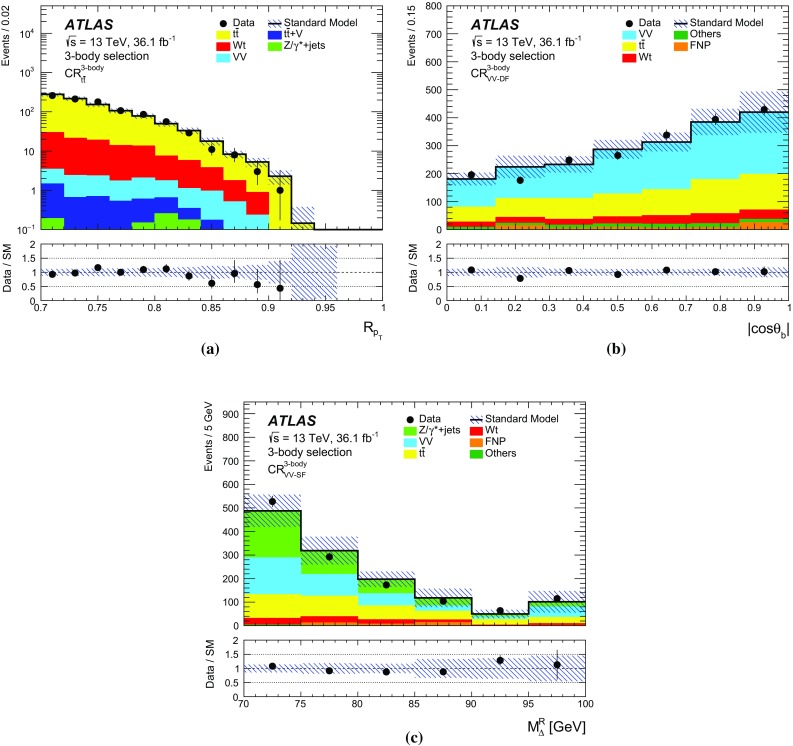
*Three-body selection* distributions of **a** $R_{p_{\text {T}}}$ in $\text {CR}^{\text {3-body}}_{t\bar{t}}$, **b** $\cos \theta _b$ in $\text {CR}^{\text {3-body}}_{VV\text {-DF}}$, and **c** $M_{\Delta }^\text {R}$ in $\text {CR}^{\text {3-body}}_{VV\text {-SF}}$ after the background fit. The contributions from all SM backgrounds are shown as a histogram stack; the hatched bands represent the total uncertainty in the background predictions after the fit to the data has been performed. The counting uncertainty on data is also shown by the black error bars. The rightmost bin of each plot includes overflow events

Table 10 shows the expected and observed numbers of events in each of the control regions after the background fit. The total number of fitted background events in the validation regions is in agreement with the observed number of data events. Figure 3 shows three distributions in the control regions after the background fit, so that the plots illustrate the MC modelling of the shape of each variable. In general, good agreement between the data and the background model is found within uncertainties. The other selection variables are equally well described by the background prediction. Good agreement, within one standard deviation from the SM background prediction, is observed in the VRs and summarised in Fig. 5.

**Table 10:** *Three-body selection* background fit results for the CRs of the SR$^{\text {3-body}}_{W}$ and SR$^{\text {3-body}}_{t}$ background fit. The nominal predictions from MC simulation, are given for comparison for those backgrounds ($t\bar{t}$, *VV*-DF and *VV*-SF) that are normalised to data in dedicated CRs. Combined statistical and systematic uncertainties are given. Entries marked “–” indicate a negligible background contribution

	$\text {CR}^{\text {3-body}}_{t\bar{t}}$	$\text {CR}^{\text {3-body}}_{VV\text {-DF}}$	$\text {CR}^{\text {3-body}}_{VV\text {-SF}}$
Observed events	951	2046	1275
Estimated SM events	$951\pm 31$	$2046\pm 50$	$1275\pm 40$
$t\bar{t}$	$833\pm 33$	$620\pm 110$	$330\pm 60$
*VV*-DF	$11.5\pm 2.4$	$1090\pm 130$	$-$
*VV*-SF	$-$	$-$	$380\pm 90$
*Wt*	$101\pm 10$	$186\pm 28$	$103\pm 17$
$t\bar{t}$ + *V*	$4.3\pm 0.4$	$0.39\pm 0.06$	$0.36\pm 0.07$
$Z/\gamma *$ + jets	$0.70\pm 0.22$	$1.8_{-1.8}^{+2.5}$	$430\pm 50$
Higgs bosons	$0.31\pm 0.08$	$79\pm 9$	$6.2\pm 0.8$
Fake and non-prompt	$0.00_{-0.00}^{+0.30}$	$65.4\pm 2.2$	$24.0\pm 1.3$
Nominal MC, $t\bar{t}$	787	590	320
Nominal MC, *VV*-DF	11.3	1069	$-$
Nominal MC, *VV*-SF	$-$	$-$	370

### Four-body selection background determination

In the four-body SR, the largest SM background contributions stem from $t\bar{t}$ and diboson production, as well as $Z/\gamma ^*+\mathrm {jets}$ production with the *Z* boson decaying into $\tau \tau$ with both $\tau$ leptons decaying leptonically. Three dedicated control regions are defined: $\text {CR}^{\text {4-body}}_{t\bar{t}}$, $\text {CR}^{\text {4-body}}_{VV}$ and $\text {CR}^{\text {4-body}}_{Z\tau \tau }$. The background predictions are tested in three validation regions that are defined to be kinematically similar to, but disjoint from, both the control and signal regions. The definitions of the control and validation regions are shown in Table 11. In the $t\bar{t}$ control region the signal contamination is less than $\sim 6\%$, while in $\text {CR}^{\text {4-body}}_{VV}$ and $\text {CR}^{\text {4-body}}_{Z\tau \tau }$ the highest signal contamination, for a top squark mass of 260 GeV and a lightest neutralino mass of 180 GeV, is respectively $\sim 30\%$ and $\sim 9\%$.

**Table 11:** *Four-body selection* control and validation regions definition. The common selection reported in Table 4 also applies to all regions

	$\text {CR}^{\text {4-body}}_{t\bar{t}}$	$\text {CR}^{\text {4-body}}_{VV}$	$\text {CR}^{\text {4-body}}_{Z\tau \tau }$	$\text {VR}^{\text {4-body}}_{t\bar{t}}$	$\text {VR}^{\text {4-body}}_{VV}$	$\text {VR}^{\text {4-body}}_{Z\tau \tau }$
Leading lepton $p_\mathrm{T}$ [GeV]	[7, 80]	[7, 80]	$> 20$	[7, 80]	[7, 80]	$> 50$
Subleading lepton $p_\mathrm{T}$ [GeV]	[7, 35]	[7, 35]	$> 20$	[7, 35]	[7, 35]	[7, 20]
$n_{\mathrm {jets}}$	$\ge 2$	$=1$	$=1$	$\ge 2$	$=1$	$=1$
Leading jet $p_\mathrm{T}$ [GeV]	[100, 150]	$> 150$	$> 150$	$> 150$	$> 150$	$> 150$
$m_{\ell \ell }$ [GeV]	$> 10$	$> 45$	[10, 45]	$> 10$	$> 45$	[10, 45]
$R_{2\ell 4j}$	–	–	–	$< 0.35$	–	–
$R_{2\ell }$	–	$< 5$	–	$< 12$	$> 5$	–
$n_{b\text {-jets}}$	–	$=0$	$=0$	–	$=0$	$=0$

**Fig. 4:**
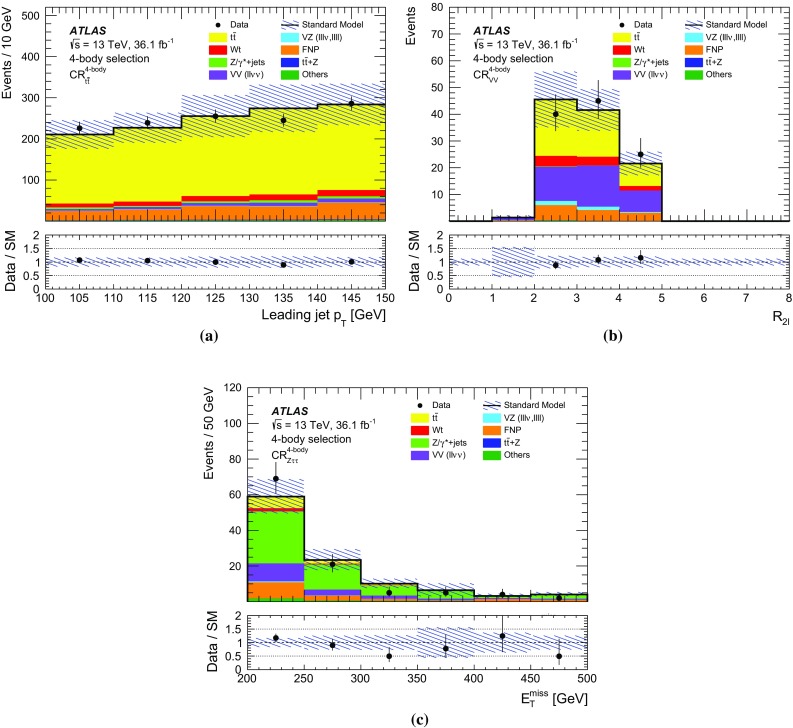
*Four-body selection* distributions of the **a** $p_\mathrm{T}(j_1)$ in $\text {CR}^{\text {4-body}}_{t\bar{t}}$, **b** $R_{2\ell }$ in $\text {CR}^{\text {4-body}}_{VV}$ and **c** $E_{\text {T}}^{\text {miss}}$ in $\text {CR}^{\text {4-body}}_{Z\tau \tau }$after the background fit. The contributions from all SM backgrounds are shown as a histogram stack; the hatched bands represent the total uncertainty in the background predictions after the fit to the data has been performed. The counting uncertainty on data is also shown by the black error bars. The rightmost bin of each plot includes overflow events

**Fig. 5:**
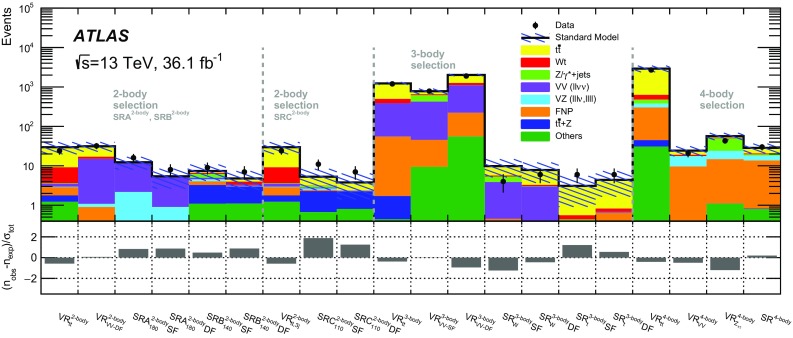
Comparison of the observed data ($n_{\mathrm{obs}}$) with the predicted SM background ($n_{\mathrm{exp}}$) in the SRs and associated VRs. The background predictions are obtained using the background-only fit configuration, and the hatched bands represent the total uncertainty in the background predictions after the fit to the data has been performed. The counting uncertainty on data is also shown by the black error bars. The bottom panel shows the difference between data and the predicted SM background divided by the total uncertainty ($\sigma _{\mathrm{tot}}$)

Table 12 shows the expected and observed numbers of events in each of the control regions after the background fit. Good agreement between data and the SM predictions is observed in the validation regions and shown in Fig. 5. Figure 4 shows three distributions in the control regions for this analysis after applying the normalisation factors provided by the background fit. Good agreement between data and the SM predictions is observed. The other selection variables are equally well described by the background prediction. The largest observed deviation ($1.4 \sigma$) from the SM background prediction is found in $\text {VR}^{\text {4-body}}_{Z\tau \tau }$. The yields in the other VRs are found to be compatible with the SM predictions within one standard deviation.

**Table 12:** *Four-body selection* background fit results for the CRs of the $\text {SR}^{\mathrm {4-body}}$ background fit. The nominal predictions from MC simulation, are given for comparison for those backgrounds ($t\bar{t}$, *VV* and $Z_{\tau \tau }$) that are normalised to data in dedicated CRs. Combined statistical and systematic uncertainties are given

	$\text {CR}^{\text {4-body}}_{t\bar{t}}$	$\text {CR}^{\text {4-body}}_{VV}$	$\text {CR}^{\text {4-body}}_{Z\tau \tau }$
Observed events	1251	110	106
Estimated SM events	$1251 \pm 35$	$110 \pm 10$	$106 \pm 10$
$t\bar{t}$	$960\pm 50$	$47 \pm 20$	$10 \pm 6$
*VV*	$37\pm 22$	$40 \pm 22$	$18 \pm 11$
$Z_{\tau \tau }$	$22\pm 8$	$0.00_{-0.00}^{+0.17}$	$54 \pm 16$
$t\bar{t}$ + Z	$5.6 \pm 0.8$	$0.08 \pm 0.01$	$0.05 \pm 0.02$
*Wt*	$62\pm 19$	$9.0 \pm 2.7$	$2.7\pm 2.4$
$Z_{ee}$, $Z_{\mu \mu }$	$0.7 \pm 0.5$	$0.2 _{-0.2}^{+0.4}$	$1.6\pm 0.6$
Others	$11.2\pm 1.6$	$0.51 \pm 0.12$	$3.2 \pm 0.6$
Fake and non-prompt	$154\pm 14$	$13.1 \pm 2.0$	$16 \pm 7$
Nominal MC,$t\bar{t}$	931	46	10
Nominal MC,*VV*	47	51	23
Nominal MC,$Z_{\tau \tau }$	20	0	51

## Systematic uncertainties

The primary sources of systematic uncertainty are related to: the jet energy scale (JES), jet energy resolution (JER), and the theoretical and MC modelling uncertainties in the backgrounds. The statistical uncertainties of the simulated event samples are also taken into account. The effect of the systematic uncertainties is evaluated for all signal samples and background processes. Since the normalisation of the dominant background processes is extracted in dedicated control regions, the systematic uncertainties only affect the extrapolation to the signal regions in these cases. Statistical uncertainties due to the limited number of data events in the CRs are also included in the fit for each region.

The JES and JER uncertainties are derived as a function of the $p_\mathrm{T}$ and $\eta$ of the jet, as well as of the pile-up conditions and the jet flavour composition of the selected jet sample [43]. Uncertainties associated to the modelling of the *b*-tagging efficiencies for *b*-jets, *c*-jets and light-flavour jets [103, 104] are also considered.

The systematic uncertainties related to the modelling of $E_{\text {T}}^{\text {miss}}$ in the simulation are estimated by propagating the uncertainties in the energy and momentum scale of electrons, muons and jets, as well as the uncertainties in the resolution and scale of the soft term [49].

Other detector-related systematic uncertainties, such as those in lepton reconstruction efficiency, energy scale, energy resolution and in the modelling of the trigger efficiency [36, 37], are found to have a small impact on the results and are generally negligible compared to the other detector-related uncertainties.

The uncertainties in the modelling of the $t\bar{t}$ and single-top backgrounds in simulation are estimated by varying the renormalisation and factorisation scales by a factor of two, as well as the amount of initial- and final-state radiation used to generate the samples [56]. Uncertainties in the parton shower modelling are assessed as the difference between the predictions from Powheg showered with Pythia and Herwig, and those due to the event generator choice by comparing Powheg and MadGraph5_aMC@NLO  [56]. An uncertainty in the acceptance due to the interference between $t\bar{t}$ and single top quark *Wt* production is assigned by comparing the predictions of dedicated LO MadGraph  2.5 samples. These samples are used to compare the predictions for $t\bar{t}$ and *Wtb* with the inclusive *WWbb* process, where the same production diagrams are included, but top quarks are not required to be on-shell.

The diboson background MC modelling uncertainties are estimated by varying up and down by a factor of two the renormalisation, factorisation and resummation scales used to generate the sample [58]. For $t\bar{t} Z$ production, the predictions from the MadGraph5_aMC@NLO and Sherpa event generators are compared and the full difference between the respective predictions is assigned as an uncertainty. Uncertainties related to the choice of renormalisation and factorisation scales are assessed by varying the corresponding event generator parameters up and down by a factor of two around their nominal values [105].

The uncertainties related to the choice of QCD renormalisation and factorisation scales in $Z/\gamma ^*+\mathrm {jets}$ events are assessed by varying the corresponding event generator parameters up and down by a factor of two around their nominal values. Uncertainties due to our choice of the resummation scale and the matching scale between the matrix element and the parton shower are estimated by varying up and down by a factor of two the corresponding parameters in Sherpa.

The cross-sections used to normalise the MC samples are varied according to the uncertainty in the cross-section calculation, i.e., 5.3% uncertainty for single top quark *Wt*-channel [106], 6% for diboson, 13% for $t\bar{t} W$ and 12% for $t\bar{t} Z$ production [61]. For $t\bar{t} WW$, *tZ*, *tWZ*, $t\bar{t} h$, $t\bar{t} t$, $t\bar{t} t\bar{t}$, and triboson production processes, which constitute a small background, a 50% uncertainty in the event yields is assumed.

Systematic uncertainties are assigned to the FNP background estimate to account for potentially different compositions (heavy flavour, light flavour or photon conversions) between the signal and control regions, as well for the contamination from prompt leptons in the regions used to measure the probabilities for loose fake or non-prompt leptons to satisfy the tight signal criteria. Parameterisations of these probabilities are independently derived from $t\bar{t}$- and multi-jet-enriched same-charge dilepton samples. The $t\bar{t}$-enriched sample is used to derive the parameterisation from which the central prediction for the FNP background is obtained. The full difference between the predictions derived from the $t\bar{t}$ and the multi-jet parameterisation is assigned as the systematic uncertainty in the central FNP prediction and symmetrised.

A 3.2% uncertainty in the luminosity measurement is also taken into consideration for all signal and background estimates that are directly derived from MC simulations.

Table 13 summarises the contributions of the different sources of systematic uncertainty in the total SM background predictions in the signal regions. The total systematic uncertainty ranges between 15% and 46%, with the dominant sources being the size of the MC event samples, the JES and $E_{\text {T}}^{\text {miss}}$ modelling, the numbers of events in the CRs and the $t\bar{t}$ theoretical uncertainties.

Theory uncertainties in the signal acceptance are taken into account. These are computed by varying the strong coupling constant $\alpha _{s}$, the renormalization and factorization scales, the CKKW scale used to match the parton shower and matrix element descriptions and the parton shower tunes. These uncertainties are mostly relevant for the four-body selection and range between 10% and 30% depending on the mass difference $m_{\tilde{t}_1}-m_{{\tilde{\chi }^0_1}}$.

**Table 13:** Sources of systematic uncertainty in the SM background estimates, estimated after the background fits. The values are given as relative uncertainties in the total expected background event yields in the SRs. Entries marked “–” indicate either a negligible contribution or an uncertainty that does not apply (for example the normalisation uncertainty for a background whose normalisation is not fitted for that specific signal region). MC statistics refer to the statistical uncertainty from the simulated event samples. The individual components can be correlated and therefore do not necessarily add up in quadrature to the total systematic uncertainty

Signal region	$\text {SRA}^{\text {2-body}}_{180}$ SF	$\text {SRA}^{\text {2-body}}_{180}$ DF	$\text {SRB}^{\text {2-body}}_{140}$ SF	$\text {SRB}^{\text {2-body}}_{140}$ DF	$\text {SRC}^{\text {2-body}}_{110}$ SF	$\text {SRC}^{\text {2-body}}_{110}$ DF	SR$^{\text {3-body}}_{W}$ SF	SR$^{\text {3-body}}_{W}$ DF	SR$^{\text {3-body}}_{t}$ SF	SR$^{\text {3-body}}_{t}$ DF	$\text {SR}^{\mathrm {4-body}}$
Total SM background uncertainty	21%	32%	15%	21%	35%	38%	36%	39%	46%	42%	20%
Diboson theoretical uncertainties	4.0%	5.9%	$-$	$-$	$-$	$-$	9.1%	10%	1.3%	$-$	2.7%
$t\bar{t}$ theoretical uncertainties	$-$	$-$	4.2%	6.6%	12%	13%	13%	18%	25%	24%	8.1%
$Wt$ theoretical uncertainties	$-$	$-$	$-$	1.9%	$-$	5.4%	$-$	$-$	$-$	$-$	$-$
$t\bar{t}$-$Wt$ interference	$-$	$-$	1.8%	7.9%	$-$	$-$	$-$	$-$	$-$	$-$	$-$
MC statistical uncertainties	13%	28%	12%	13%	15%	15%	16%	14%	20%	22%	10%
*VV* normalisation	14%	$-$	$-$	$-$	$-$	$-$	12%	4.3%	1.3%	$-$	9.2%
$t\bar{t}$ normalisation	$-$	$-$	$-$	$-$	16%	15%	1.8%	2.5%	3.5%	3.5%	8.6%
$t\bar{t} + Z$ normalisation	$-$	$-$	7.6%	9.9%	8.5%	10%	$-$	$-$	$-$	$-$	$-$
$Z_{\tau \tau }$ normalisation	$-$	$-$	$-$	$-$	$-$	$-$	$-$	$-$	$-$	$-$	1.5%
Jet energy scale	6.9%	3.1%	4.1%	6.4%	13%	22%	19%	18%	27%	11%	4.4%
Jet energy resolution	$-$	$-$	$-$	$-$	12%	16%	7.2%	18%	2.9%	22%	1.0%
$E_{\text {T}}^{\text {miss}}$ modelling	5.0%	13%	2.2%	3.2%	26%	23%	18%	11%	14%	6.5%	1.3%
$b$-tagging	$-$	$-$	3.0%	1.5%	$-$	$-$	2.7%	3.0%	1.0%	3.0%	2.2%
Pile-up reweighting	2.0%	3.2%	1.1%	4.3%	2.9%	4.6%	2.9%	5.0%	5.1%	4.9%	1.4%
Lepton modelling	1.3%	2.1%	$-$	1.1%	$-$	$-$	1.1%	3.1%	4.6%	3.0%	2.5%
Fake and non-prompt leptons	$-$	$-$	7.4%	$-$	4.0%	$-$	2.8%	$-$	$-$	$-$	14%

## Results

The data are compared to background predictions in the signal regions of the different selections. The number of observed events and the predicted number of SM background events from the background-only fits in all SRs and VRs are shown in Fig. 5. In all SRs, good agreement is observed between data and the SM background predictions. A detailed discussion of the results is given in the following sections.

### Two-body results

**Fig. 6:**
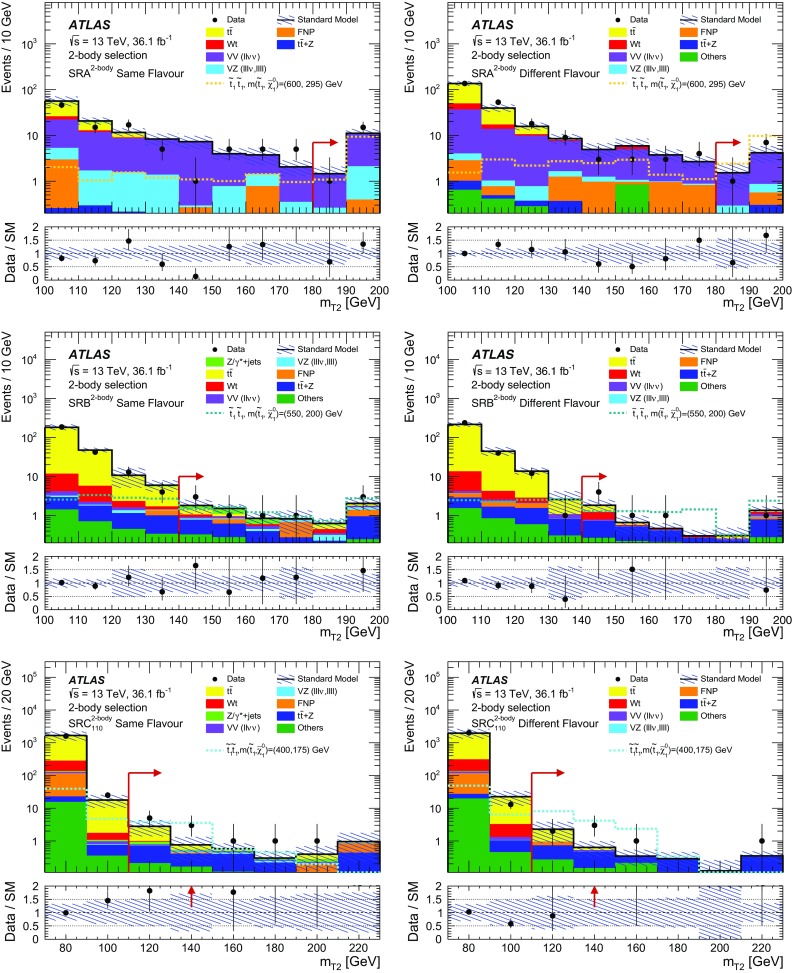
*Two-body selection* distributions of $m_{\tiny {\text{ T2 }}}^{\ell \ell }$ for events satisfying the selection criteria of the six SRs, except for the one on $m_{\tiny {\text{ T2 }}}^{\ell \ell }$, after the background fit. The contributions from all SM backgrounds are shown as a histogram stack; the hatched bands represent the total uncertainty in the background predictions after the fit to the data has been performed. The counting uncertainty on data is also shown by the black error bars. The rightmost bin of each plot includes overflow events. Reference top squark pair production signal models are overlayed for comparison. Red arrows indicate the signal region selection criteria

Figure 6 shows the $m_{\tiny {\text{ T2 }}}^{\ell \ell }$ distribution in each of the two-body signal regions, split between the same- and different-flavour lepton channels, omitting the selection on $m_{\tiny {\text{ T2 }}}^{\ell \ell }$ itself. The estimated SM yields in $\text {SRA}^{\text {2-body}}_{180}$ and $\text {SRB}^{\text {2-body}}_{140}$ are determined with a background fit simultaneously determining the normalisations of the background contributions from $t\bar{t}$, diboson with a SF lepton pair, $t\bar{t} + Z$ and diboson with more than two charged leptons by including $\text {CR}^{\text {2-body}}_{t\bar{t}}$, $\text {CR}^{\text {2-body}}_{VV\text {-SF}}$, $\text {CR}^{\text {2-body}}_{t\bar{t} Z}$ and $\text {CR}^{\text {2-body}}_{VZ}$ in the likelihood minimisation. The estimated SM yields in $\text {SRC}^{\text {2-body}}_{110}$ are determined with a background fit simultaneously determining the normalisations of the background contributions from $t\bar{t}$ and $t\bar{t} + Z$ by including $\text {CR}^{\text {2-body}}_{t\bar{t},3j}$ and $\text {CR}^{\text {2-body}}_{t\bar{t} Z}$ in the likelihood minimisation. No significant excess over the SM prediction is observed, as can be seen from the background-only fit results which are shown in Table 14 for $\text {SRA}^{\text {2-body}}_{180}$ and $\text {SRB}^{\text {2-body}}_{140}$, and Table 15 for the $\text {SRC}^{\text {2-body}}_{110}$. Table 16 reports the observed and expected yields for the SRs used for the computation of the exclusion limits.

**Table 14:** *Two-body selection* background fit results for $\text {SRA}^{\text {2-body}}_{180}$ and $\text {SRB}^{\text {2-body}}_{140}$. The nominal predictions from MC simulation, are given for comparison for those backgrounds ($t\bar{t}$, *VV*-SF, $t\bar{t} Z$ and *VZ*) that are normalised to data in dedicated CRs. The “Others” category contains the contributions from $t\bar{t} W$, $t\bar{t} h$, $t\bar{t} WW$, $t\bar{t} t$, $t\bar{t} t\bar{t}$, *Wh*, *ggh* and *Zh* production. Combined statistical and systematic uncertainties are given. Entries marked “–” indicate a negligible background contribution. The “Others” contribution to $\text {SRB}^{\text {2-body}}_{140}$ is dominated by $t\bar{t} W$

	$\text {SRA}^{\text {2-body}}_{180}$ SF	$\text {SRA}^{\text {2-body}}_{180}$ DF	$\text {SRB}^{\text {2-body}}_{140}$ SF	$\text {SRB}^{\text {2-body}}_{140}$ DF
Observed events	16	8	9	7
Estimated SM events	$12.3 \pm 2.6$	$5.4 \pm 1.7$	$7.4 \pm 1.1$	$4.8 \pm 1.0$
$t\bar{t}$	$-$	$-$	$0.8 \pm 0.4$	$0.8 \pm 0.5$
*Wt* events	$-$	$-$	$0.38 \pm 0.29$	$0.7 \pm 0.5$
$Z/\gamma ^*+\mathrm {jets}$	$0.35 \pm 0.21$	$-$	$1.24 \pm 0.32$	$0.03 \pm 0.01$
Fake and non-prompt	$0.00_{-0.00}^{+0.30}$	$0.00_{-0.00}^{+0.30}$	$0.8 \pm 0.5$	$0.00_{-0.00}^{+0.30}$
*VV*-DF	$-$	$4.5 \pm 1.5$	$-$	$0.23 \pm 0.06$
*VV*-SF	$9.8 \pm 2.5$	$-$	$0.39 \pm 0.11$	$-$
*VZ*	$1.91 \pm 0.31$	$0.52 \pm 0.17$	$0.53 \pm 0.14$	$0.04 \pm 0.01$
$t\bar{t}$ + *Z*	$0.08 \pm 0.03$	$0.15 \pm 0.06$	$2.3 \pm 0.6$	$1.8 \pm 0.5$
Others	$0.18 \pm 0.02$	$0.24 \pm 0.07$	$1.10 \pm 0.16$	$1.11 \pm 0.16$
Nominal MC, $t\bar{t}$	$-$	$-$	0.78	0.8
Nominal MC, *VV*-SF	8.8	$-$	0.35	$-$
Nominal MC, *VZ*	1.9	0.52	0.54	0.04
Nominal MC, $t\bar{t} + Z$	0.09	0.17	2.6	2.2

**Table 15:** *Two-body selection* background fit results for $\text {SRC}^{\text {2-body}}_{110}$. The nominal predictions from MC simulation, are given for comparison for those backgrounds ($t\bar{t}$ and $t\bar{t} Z$) that are normalised to data in dedicated CRs. The “Others” category contains the contributions from $t\bar{t} W$, $t\bar{t} h$, $t\bar{t} WW$, $t\bar{t} t$, $t\bar{t} t\bar{t}$, *Wh*, *ggh* and *Zh* production. Combined statistical and systematic uncertainties are given. Entries marked “–” indicate a negligible background contribution

	$\text {SRC}^{\text {2-body}}_{110}$ SF	$\text {SRC}^{\text {2-body}}_{110}$ DF
Observed events	11	7
Estimated SM events	$5.3 \pm 1.8$	$3.8 \pm 1.5$
$t\bar{t}$	$2.1\pm 1.3$	$1.4\pm 1.2$
$t\bar{t}$ + Z	$1.6 \pm 0.5$	$1.4 \pm 0.5$
*Wt*	$0.05_{-0.05}^{+0.09}$	$0.00_{-0.00}^{+0.23}$
*VV* + *VZ*	$0.33 \pm 0.06$	$0.12 \pm 0.04$
$Z/\gamma ^*+\mathrm {jets}$	$0.3_{-0.3}^{+0.5}$	$-$
Others	$0.67 \pm 0.13$	$0.81 \pm 0.15$
Fake and non-prompt	$0.18_{-0.18}^{+0.41}$	$0.00_{-0.00}^{+0.02}$
Nominal MC, $t\bar{t}$	2.3	1.6
Nominal MC, $t\bar{t} + \hbox {Z}$	1.9	1.70

**Table 16:** *Two-body selection* background fit results for $\mathrm {SR(A,B)}^{\mathrm {2-body}}_{x,y}$ regions, where *x* and *y* denote the low and high edges of the bin. Combined statistical and systematic uncertainties are given. Uncertainties in the predicted background event yields are quoted as being symmetric

	Lepton flavour	$\mathrm {SRA}^{\mathrm {2-body}}_{120,140}$	$\mathrm {SRB}^{\mathrm {2-body}}_{120,140}$	$\mathrm {SRA}^{\mathrm {2-body}}_{140,160}$	$\mathrm {SRA}^{\mathrm {2-body}}_{160,180}$
Observed events	SF	22	17	6	10
Estimated SM events		$20.0 \pm 4.6$	$16.3 \pm 6.2$	$11.0 \pm 2.5$	$5.6 \pm 1.8$
Observed events	DF	27	13	6	7
Estimated SM events		$23.8 \pm 4.2$	$16.1 \pm 5.3$	$10.8 \pm 2.1$	$6.4 \pm 1.3$

### Three-body results

**Fig. 7:**
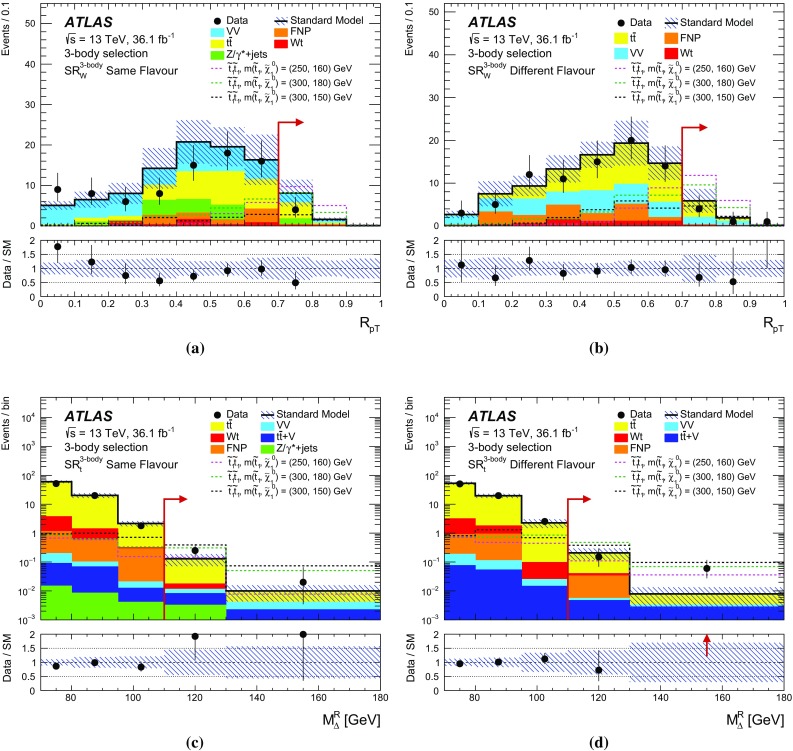
*Three-body selection* distributions of $R_{p_{\text {T}}}$ in **a** same-flavour and **b** different-flavour events that satisfy all the SR$^{\text {3-body}}_{W}$ selection criteria except for the one on $R_{p_{\text {T}}}$, and of $M_{\Delta }^\text {R}$ in the **c** same-flavour and **d** different-flavour events that satisfy all the SR$^{\text {3-body}}_{t}$ selection criteria except for the one on $M_{\Delta }^\text {R}$ after the background fit. The contributions from all SM backgrounds are shown as a histogram stack; the hatched bands represent the total uncertainty in the background predictions after the fit to the data has been performed. The counting uncertainty on data is also shown by the black error bars. The rightmost bin of each plot includes overflow events. Reference top squark pair production signal models are overlayed for comparison. Red arrows indicate the signal region selection criteria

Figure 7 shows the distributions of $R_{p_{\text {T}}}$ and $M_{\Delta }^\text {R}$ in each of the signal regions, split between the same- and different-flavour channels, omitting the requirement on $R_{p_{\text {T}}}$ and on $M_{\Delta }^\text {R}$. The estimated SM yields in SR$^{\text {3-body}}_{W}$ and SR$^{\text {3-body}}_{t}$ are determined with a background fit simultaneously determining the normalisations of $t\bar{t}$, SF diboson production and DF diboson production by including $\text {CR}^{\text {3-body}}_{t\bar{t}}$, $\text {CR}^{\text {3-body}}_{VV\text {-SF}}$ and $\text {CR}^{\text {3-body}}_{VV\text {-DF}}$ in the likelihood minimisation. No excess over the SM prediction is observed. Table 17 shows the background fit results.

**Table 17:** *Three-body selection* background fit results for SR$^{\text {3-body}}_{W}$ and SR$^{\text {3-body}}_{t}$. The nominal predictions from MC simulation, are given for comparison for those backgrounds ($t\bar{t}$, *VV*-DF and *VV*-SF) that are normalised to data in dedicated CRs. Combined statistical and systematic uncertainties are given. Entries marked “–” indicate a negligible background contribution

	SR$^{\text {3-body}}_{W}$ SF	SR$^{\text {3-body}}_{W}$ DF	SR$^{\text {3-body}}_{t}$ SF	SR$^{\text {3-body}}_{t}$ DF
Observed events	4	6	6	6
Estimated SM events	$9.8\pm 3.4$	$7.8\pm 3.0$	$3.1\pm 1.4$	$4.4\pm 1.8$
$t\bar{t}$	$4.2\pm 1.6$	$4.6\pm 2.1$	$2.5\pm 1.3$	$3.6\pm 1.8$
*VV*-DF	$-$	$2.9\pm 1.4$	$-$	$0.04\pm 0.03$
*VV*-SF	$3.4\pm 2.1$	$-$	$0.16\pm 0.08$	$-$
*Wt*	$0.31\pm 0.22$	$0.23\pm 0.12$	$0.12\pm 0.05$	$0.14\pm 0.08$
$t\bar{t} + V$	$0.03\pm 0.01$	$0.06\pm 0.02$	$0.18\pm 0.04$	$0.24\pm 0.07$
$Z/\gamma ^*$ + jets	$1.5\pm 0.7$	$0.05\pm 0.01$	$0.1\pm 0.03$	$0.0\pm 0.0$
Fake and non-prompt	$0.42\pm 0.28$	$0.06\pm 0.06$	$0.00_{-0.00}^{+0.30}$	$0.41\pm 0.09$
Nominal MC, $t\bar{t}$	4.0	4.3	2.4	3.4
Nominal MC, *VV*-DF	$-$	2.8	$-$	0.04
Nominal MC, *VV*-SF	3.4	$-$	0.16	$-$

### Four-body results

**Fig. 8:**
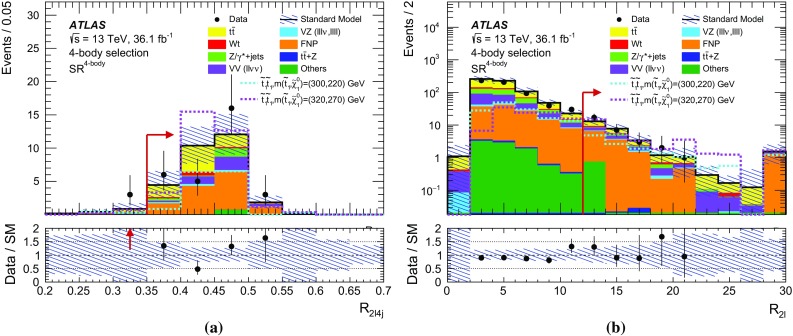
*Four-body selection* distributions of **a** $R_{2\ell 4j}$ and **b** $R_{2\ell }$ for events satisfying all the $\text {SR}^{\mathrm {4-body}}$ selections except for the one on the variable shown in the figure, after the background fit. The contributions from all SM backgrounds are shown as a histogram stack; the hatched bands represent the total uncertainty in the background predictions after the fit to the data has been performed. The counting uncertainty on data is also shown by the black error bars. The rightmost bin of each plot includes overflow events. Reference top squark pair production signal models are overlayed for comparison. Red arrows indicate the signal region selection criteria

Figure 8 shows the distributions of $R_{2\ell 4j}$ and $R_{2\ell }$ for events satisfying all the $\text {SR}^{\mathrm {4-body}}$ selections. No significant excess over the SM prediction is visible. The estimated SM yields in $\text {SR}^{\mathrm {4-body}}$ are determined with a background fit simultaneously determining the normalisations of $t\bar{t}$, diboson production, and $Z/\gamma ^*+\mathrm {jets}$ where $Z \rightarrow \tau \tau$, by including $\text {CR}^{\text {4-body}}_{t\bar{t}}$, $\text {CR}^{\text {4-body}}_{VV}$ and $\text {CR}^{\text {4-body}}_{Z\tau \tau }$ in the likelihood minimisation. The background fit results are shown in Table 18. The observed yield is less than one standard deviation from the background prediction in the SR.

**Table 18:** *Four-body selection* background fit results for $\text {SR}^{\mathrm {4-body}}$. The nominal predictions from MC simulation, are given for comparison for those backgrounds ($t\bar{t}$, VV and $Z_{\tau \tau }$) that are normalised to data in dedicated CRs. The “Others” category contains the contributions from $t\bar{t} W$, $t\bar{t}h$, $t\bar{t}WW$, $t\bar{t}t$, $t\bar{t}t\bar{t}$, *Wh*, *ggh* and *Zh* production. Combined statistical and systematic uncertainties are given

	$$\text {SR}^{\mathrm {4-body}}$$
Observed events	30
Estimated SM events	$28 \pm 6$
$t\bar{t}$	$7.9 \pm 2.0$
*VV*	$4.5 \pm 2.3$
$Z_{\tau \tau }$	$1.2 \pm 0.6$
$t\bar{t} + \hbox {Z}$	$0.03\pm 0.01$
*Wt*	$1.08 \pm 0.27$
$Z_{ee}$, $Z_{\mu \mu }$	$0.21 \pm 0.09$
Others	$0.80 \pm 0.30$
Fake and non-prompt	$12.8 \pm 4.3$
Nominal MC, $t\bar{t}$	7.7
Nominal MC, *VV*	5.7
Nominal MC, $Z_{\tau \tau }$	1.1

### Interpretation

Two different sets of exclusion limits are derived for models of new physics beyond the SM. A model-independent upper limit on the visible cross-section $\sigma _{\mathrm {vis}}$ of new physics, defined as the ratio between the upper limit at 95% CL on the number of signal events $S^{95}$ and the integrated luminosity, is derived in each SR by performing a fit which includes the observed yield in the SR as a constraint, and a free signal yield in the SR as an additional process. The CL$_\mathrm {s}$ method [107] is used to derive all the exclusion confidence levels. These limits assume negligible signal contamination in the CRs. This assumption leads to conservative results when comparing with model-dependent limits for models that predict a sizeable contamination in the CRs. Model-independent upper limits are presented in Table 19.

**Table 19:** Model-independent 95% CL upper limits on the visible cross-section ($\sigma _{\mathrm {vis}}$) of new physics, the visible number of signal events ($S^{95}_{\text {o}bs}$), the visible number of signal events ($S^{95}_{\text {e}xp}$) given the expected number of background events (and $\pm 1\sigma$ excursions of the expected number), and the discovery *p*-value ($p(s = 0)$), all calculated with pseudo-experiments, are shown for each SR

	Signal region	$\sigma _{\mathrm {vis}}$ [fb]	$S_{\text {o}bs}^{95}$	$S_{\text {e}xp}^{95}$	$p(s=0)$
Two-body	$\text {SRA}^{\text {2-body}}_{180}$ SF	0.37	13.2	${10}^{+4}_{-3}$	0.20
	$\text {SRA}^{\text {2-body}}_{180}$ DF	0.26	9.5	${7.0}^{+3.0}_{-1.8}$	0.19
	$\text {SRB}^{\text {2-body}}_{140}$ SF	0.24	8.6	${7.2}^{+2.7}_{-1.8}$	0.28
	$\text {SRB}^{\text {2-body}}_{140}$ DF	0.23	8.4	${6.0}^{+2.7}_{-1.3}$	0.19
	$\text {SRC}^{\text {2-body}}_{110}$ SF	0.36	13.0	$7.4^{+3.1}_{-2.0}$	0.05
	$\text {SRC}^{\text {2-body}}_{110}$ DF	0.26	9.5	$6.3^{+2.5}_{-1.6}$	0.12
Three-body	SR$^{\text {3-body}}_{W}$-SF	0.17	6.1	${9}^{+4}_{-2}$	0.72
	SR$^{\text {3-body}}_{W}$-DF	0.21	7.5	${8.5}^{+3.5}_{-2.0}$	0.85
	SR$^{\text {3-body}}_{t}$-SF	0.24	8.8	${6.0}^{+2.4}_{-1.4}$	0.12
	SR$^{\text {3-body}}_{t}$-DF	0.23	8.2	${6.6}^{+2.8}_{-1.6}$	0.28
Four-body	$\text {SR}^{\mathrm {4-body}}$	0.48	17.4	$16^{+7}_{-5}$	0.37

Model-dependent limits are computed for various $\tilde{t}_1$ pair production scenarios. Profile likelihood fits are performed including the expected signal yield and its associated uncertainties in the CRs and SRs. All limits are quoted at 95% CL. When setting limits, the regions included in the $m_{\tiny {\text{ T2 }}}^{\ell \ell }$ shape fits ($\mathrm {SRA}^{\mathrm {2-body}}_{x,y}$ and $\mathrm {SRB}^{\mathrm {2-body}}_{x,y}$) are statistically combined. Similarly, the SR$^{\text {3-body}}_{W}$ and SR$^{\text {3-body}}_{t}$ signal regions are statistically combined as well. For each signal model, the SR with the best expected limit is used for setting the final limit.

**Fig. 9:**
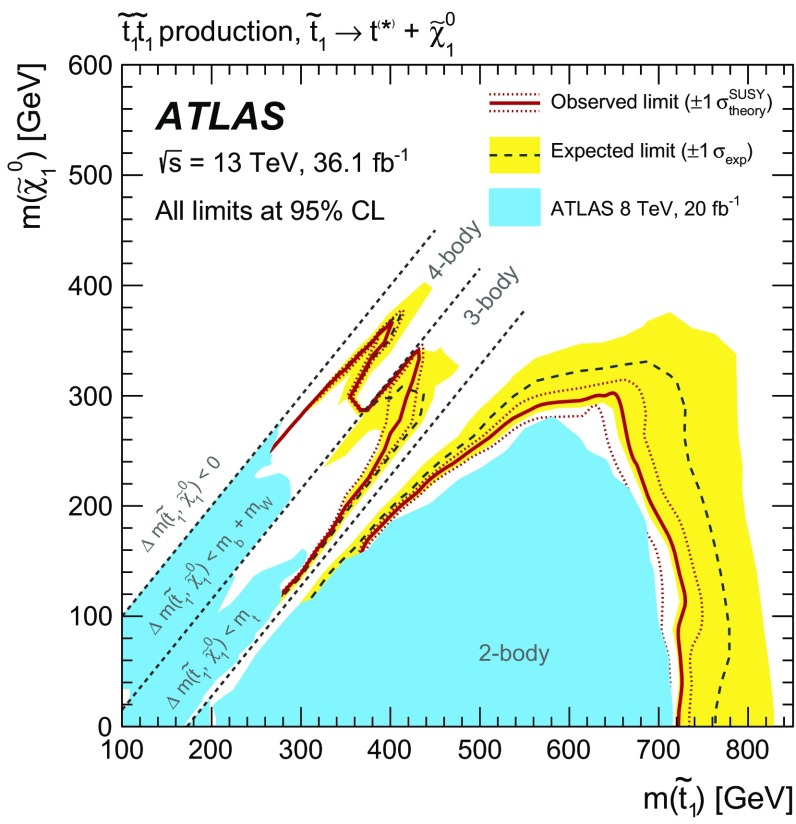
Exclusion contour for a simplified model assuming $\tilde{t}_1$ pair production, decaying via $\tilde{t}_1 \rightarrow t^{(*)}{\tilde{\chi }^0_1}$ with 100% branching ratio. The dashed grey line and the shaded yellow band are the expected limit and its $\pm 1\sigma$ uncertainty. The thick solid red line is the observed limit for the central value of the signal cross-section. The expected and observed limits do not include the effect of the theoretical uncertainties in the signal cross-section. The dotted lines show the effect on the observed limit when varying the signal cross-section by $\pm 1\sigma$ of the theoretical uncertainty. The shaded blue areas show the observed exclusion from the ATLAS $\sqrt{s}=8$ TeV analyses [18]

Limits for simplified models in which pair-produced $\tilde{t}_1$ decay with 100% branching ratio into a top quark and $\tilde{\chi }^0_1$ are shown in the $\tilde{t}_1$–$\tilde{\chi }^0_1$ mass plane in Fig. 9. The various SRs cover the different $\tilde{t}_1$ mass ranges, as described in Table 1. Top squark masses up to 720 GeV are excluded for a massless lightest neutralino. Neutralino masses up to 300 GeV are excluded for $m_{\tilde{t}_1}=645$ GeV. In the three-body decay hypothesis, top squark masses are excluded up to 430 GeV for $m_{\tilde{t}_1}-m_{{\tilde{\chi }^0_1}}$ close to the *W* boson mass. In the four-body decay hypothesis, top squark masses are excluded up to 400 GeV for $m_{\tilde{t}_1}-m_{{\tilde{\chi }^0_1}}=40$ GeV.

**Fig. 10:**
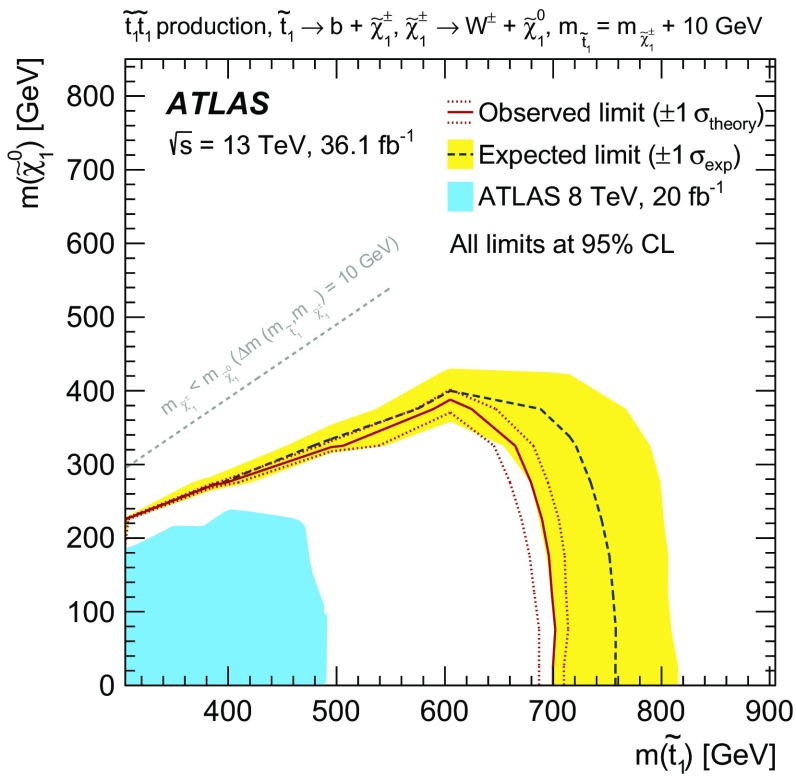
Exclusion contour for a simplified model assuming $\tilde{t}_1$ pair production, decaying via $\tilde{t}_1 \rightarrow b{\tilde{\chi }^\pm _1}$ with 100% branching ratio. The lightest chargino mass is assumed to be close to the stop mass, $m_{{\tilde{\chi }^\pm _1}} = m_{\tilde{t}_1}-10$ GeV. The dashed grey line and the shaded yellow band are the expected limit and its $\pm 1\sigma$ uncertainty. The thick solid red line is the observed limit for the central value of the signal cross-section. The expected and observed limits do not include the effect of the theoretical uncertainties in the signal cross-section. The dotted lines show the effect on the observed limit when varying the signal cross-section by $\pm 1\sigma$ of the theoretical uncertainty. The shaded blue area shows the observed exclusion from the ATLAS $\sqrt{s}=8$ TeV analysis [18]

Limits are shown for a class of simplified models in which only pair-produced $\tilde{t}_1$ decaying with 100% branching ratio into the lightest chargino and a *b*-quark are considered. Figure 10 shows the interpretation in the $\tilde{t}_1$–$\tilde{\chi }^0_1$ mass plane assuming that $m_{\tilde{t}_1} - m_{{\tilde{\chi }^\pm _1}} = 10$ GeV. Top squark masses up to 700 GeV are excluded for an LSP mass up to 200 GeV.

**Fig. 11:**
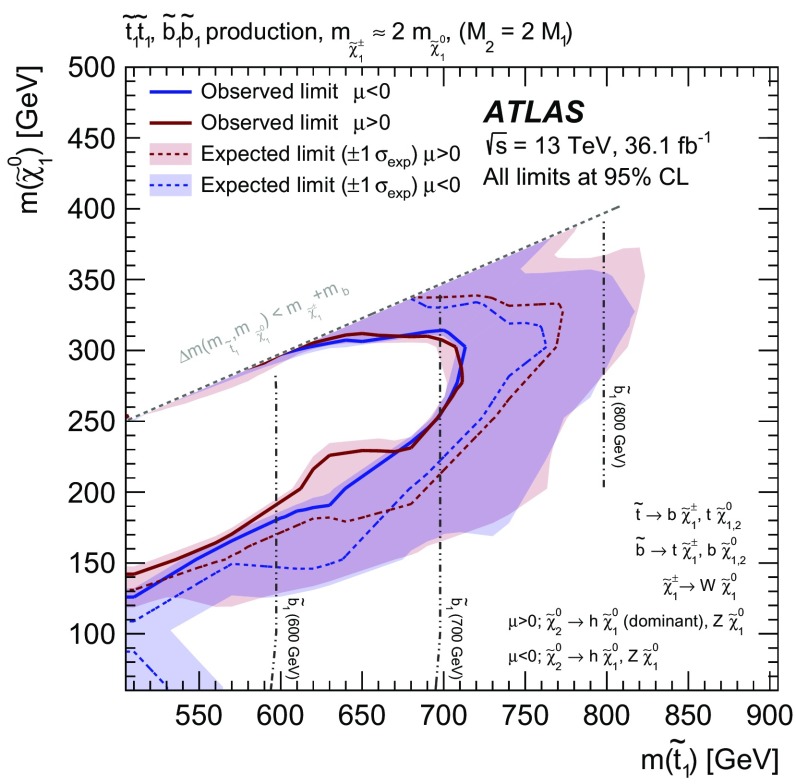
Exclusion contour as a function of $m_{\tilde{t}_1}$ and $m_{{\tilde{\chi }^0_1}}$ in the pMSSM model described in the text. Pair production of $\tilde{t}_1$ and $\tilde{b}_1$ are considered. Limits are set for both the positive (red in the figure) and negative (blue in the figure) values of $\mu$. The dashed and dotted grey lines indicate constant values of the $\tilde{b}_1$ mass. The signal models included within the shown contours are excluded at 95% CL. The dashed lines and the shaded band are the expected limit and its $\pm 1\sigma$ uncertainty. The thick solid line is the observed limit for the central value of the signal cross-section. The expected and observed limits do not include the effect of the theoretical uncertainties in the signal cross-section

Finally, limits are set on a pMSSM model where the wino and bino mass parameters, $M_1$ and $M_2$, are set to $M_2 = 2M_1$ and $m_{\tilde{t}_1} > m_{{\tilde{\chi }^\pm _1}}$. The remaining pMSSM parameters [16, 17] have the following values: $M_3 = 2.2$ TeV (gluino mass parameter), $M_{S} = \sqrt{m_{\tilde{t}_1} m_{\tilde{t}_2}} = 1.2$ TeV (product of top squark masses), $X_{t}/M_{S} = \sqrt{6}$ (mixing parameter between the left- and right-handed states), and $\tan {\beta } = 20$ (ratio of vacuum expectation values of the two Higgs doublets). The values of $M_3$ and $M_{S}$ have been chosen in order to avoid the current gluino and top squark mass limits, while the value of $X_{t}/M_{S}$ is assumed to obtain a low-mass lightest top squark while maintaining the models consistent with the observed Higgs boson mass of 125 GeV. Limits are set for both the positive and negative values of $\mu$ (the Higgs mass parameter) as a function of $m_{\tilde{t}_1}$ and $m_{{\tilde{\chi }^0_1}}$, and are shown in Fig. 11. Top squark masses up to about 700 GeV are excluded for a lightest neutralino of about 280 GeV. The sensitivity for low values of $m_{{\tilde{\chi }^0_1}}$ is limited by the $m_{\tiny {\text{ T2 }}}^{\ell \ell }$ selection acceptance, since $m_{{\tilde{\chi }^\pm _1}} - m_{{\tilde{\chi }^0_1}}$ is reduced by assuming $M_2 = 2M_1$.

## Conclusion

This article reports a search for direct top squark pair production in final states containing two opposite-charge leptons and large missing transverse momentum, based on a $36.1~\hbox {fb}^{-1}$ dataset of $\sqrt{s} = 13$ TeV proton–proton collisions recorded by the ATLAS experiment at the LHC in 2015 and 2016. Good agreement was found between the observed events in the data and the expected Standard Model yields.

Model-independent 95% CL upper limits on the visible cross-section for new phenomena were computed. The results are also interpreted in terms of simplified models assuming a range of top squark and lightest neutralino masses, with the former decaying into the latter via either a direct two-, three- or four-body decay or via an intermediate chargino state. In the case of top squark decays into $t^{(*)}{\tilde{\chi }^0_1}$, top squark masses below 720 GeV are excluded for a massless lightest neutralino. In the three-body decay hypothesis, top squark masses are excluded up to 430 GeV for $m_{\tilde{t}_1}-m_{{\tilde{\chi }^0_1}}$ close to the *W* boson mass. In the four-body decay hypothesis, top squark masses are excluded up to 400 GeV for $m_{\tilde{t}_1}-m_{{\tilde{\chi }^0_1}}=40$ GeV. Both these results extend the coverage of previous searches by about 100 GeV. The chargino decay mode, $\tilde{t}_1 \rightarrow b {\tilde{\chi }^\pm _1}$, is excluded for top squark masses up to 700 GeV, assuming that $m_{\tilde{t}_1} - m_{{\tilde{\chi }^\pm _1}} = 10$ GeV, extending the previous results by almost 200 GeV. When considering a pMSSM-inspired model including multiple decay chains, top squark masses up to about 700 GeV are excluded for a lightest neutralino of about 280 GeV. These results extend the region of supersymmetric parameter space excluded by previous LHC searches.

## Footnotes

1 ATLAS uses a right-handed coordinate system with its origin at the nominal interaction point (IP) in the centre of the detector and the *z*-axis along the beam pipe. The *x*-axis points from the IP to the centre of the LHC ring, and the *y*-axis points upwards. Cylindrical coordinates $(r,\phi )$ are used in the transverse plane, $\phi$ being the azimuthal angle around the *z*-axis. The pseudorapidity is defined in terms of the polar angle $\theta$ as $\eta = -\ln \tan (\theta /2)$. Rapidity is defined as $y=0.5 \ln \left[ \left( E + p_{z} \right) / \left( E - p_{z} \right) \right]$ where *E* denotes the energy and $p_{z}$ is the component of the momentum along the beam direction.

2 The transverse mass is defined by the equation $m_T (\mathbf {p}_{\mathrm {T}}, \mathbf {q}_{\mathrm {T}})=\sqrt{2|\mathbf {p}_{\mathrm {T}}||\mathbf {q}_{\mathrm {T}}|(1-\mathrm {cos}(\Delta \phi ))}$, where $\Delta \phi$ is the angle between the particles of negligible mass with transverse momenta $\mathbf {p}_{\mathrm {T}}$ and $\mathbf {q}_{\mathrm {T}}$.
